# Impact of level of nutritional dose and diet specific components of colostrum in promoting 24 h gain, circulating lipid profile, and circulating levels of immunocrit, proteins, glucose, and free amino acids in neonatal gilt piglets

**DOI:** 10.1371/journal.pone.0341179

**Published:** 2026-01-27

**Authors:** Linda M. Beckett, Wonders Ogundare, Leriana Garcia Reis, KaLynn Harlow, Addison Hill, Michayla Dinn, Emma Shelton, Evy Tobolski, Caeden Meade, Yu Han-Hallett, Christina Ramires Ferreira, Kara Stewart, Theresa M. Casey

**Affiliations:** 1 Purdue University Department of Animal Sciences, West Lafayette, Indiana, United States of America; 2 Animal Biosciences and Biotechnology Laboratory, Beltsville Agricultural Research Center, Agricultural Research Service, United States Department of Agriculture, Beltsville, Maryland, United States of America; 3 Purdue University Bindley Bioscience Center, Metabolite Profiling Facility, West Lafayette, Indiana, United States of America; Sun Yat-Sen University, CHINA

## Abstract

Colostrum (COL) intake relates to growth trajectory of pigs, which may be driven by differences in nutrient level, bioactive factors, or both. This study’s objective was to determine the effect of feeding COL versus milk replacer (MR) at high and low doses [20% or 10% of birth body weight (BBW)] on 24 h weight gain, body temperature, circulating levels of glucose, protein, immunocrit, free amino acids, lipids and small metabolites. At birth, gilt piglets (n = 57) were assigned to: pooled COL fed at 20% (COL20) or 10% of BBW (COL10); MR fed at 20% (MR20) or 10% of BBW (MR10); stay on sow (SOS) to suckle COL; or immediately euthanized at birth (zero hour; ZH). COL20, COL10, MR20, and MR10 were bottle fed diets every 2 h for 24 h, then piglets were euthanized, and blood collected. Serum samples were analyzed for glucose and protein using a colorimetric assay, immunocrit using the immunocrit ratio, free amino acids using liquid chromatography mass spectrometry, and lipidome using multiple reaction monitoring profiling. Dose affected glucose (*P* = 0.02), being greater in 20% treatments than 10%. Diet and dose impacted immunocrit (*P* < 0.0001) and protein (*P* = 0.04), being highest in COL20, intermediate in COL10, and lowest in MR treatments. Diet type impacted all free amino acids (*P* < 0.05). Diet and dose impacted concentration of triacylglycerols (*P* = 0.0003), cholesterol esters (diet *P* = 0.001; dose *P* = 0.05), and only diet impacted phospholipids (*P* = 0.04). Diet impacted individual triacylglycerol profile, with fatty acids longer and more unsaturated in COL than MR. Piglets on SOS, COL20, MR20 gained weight and maintained rectal temperature, whereas COL10 and MR10 lost weight and had lower temperatures (*P* < 0.05). These data and previous studies indicate colostral proteins remain intact post-absorption preventing use for energy. Due to relatively high lactose in MR and circulating metabolite profiles, we postulated piglets fed MR prioritized glycolysis to support growth, whereas COL-fed depended on fatty acid oxidation. Further studies are warranted to understand how absorptive and post-absorptive metabolism respond to diet type and dose and contribute to circulating profiles of nutrients and metabolites.

## Introduction

The lactation phase of swine production impacts neonate health, growth at weaning and long-term productive and reproductive performance of swine. Colostrum (COL) is the first milk available to neonates after birth and varying levels of intake the first 24 h postnatal relate to differential growth trajectories and fertility of swine [1,2]. Varying effects are likely due to differential intake of nutrients in COL like lipids and branched chain amino acids as well as non-nutritional bioactive factors like immunomodulatory cytokines, hormones, and maternally derived immune cells, and antibodies. Low COL intake of gilts links to poor reproductive and lactation performance as a production sow [3,4]. Low or no intake of COL leads to accumulation of lipid substrates in tissues that are catabolized in peroxisomes, including very long chain fatty acids. Lipid accumulation in vaginal tissue of gilts at 3 weeks of age linked to their infertility when they reach maturity [5]. Together data indicate that low or no intake of colostrum impacts lipid metabolism and may be linked to long-term phenotype of swine.

Colostrum plays a central role in mediating the metabolic transition of neonates to the external environment. Upon birth, neonates need to transition metabolically from a continuous high-carbohydrate intravenous source of nutrition to intermittent oral intake of milk, which provides 50% of its kilocalories as fat [6]. To utilize milk fats for energy, metabolic and cellular signaling pathways need to be initiated. Postnatal intake of colostrum appears to have a unique ability to increase fatty acid oxidation, ketogenesis (acetogenesis in swine) and biogenesis of lipid metabolizing peroxisomes [7–11] that cannot be replicated by feeding milk replacer (MR) formula nor mature milk, which is produced by sows beginning on lactation day 10 [1]. Therefore, our finding of relative accumulation of peroxisomal lipid substrates in tissues, likely indicate low or no intake of COL results in inadequate activation of fatty acid oxidation [5].

The stage of morphological and physiological development of the neonate at birth also plays a role in its metabolic response to colostrum [3,4]. At birth, fetal-type enterocytes line the piglet’s gut, and appear to be capable of taking up entire proteins and other macromolecules for transmission of them into circulation [12,13], with colostrum intake inducing the transmission process [13,14]. The transmission of whole proteins from the neonate’s intestinal lumen into circulation potentially enables maintenance of their bioactivity and may explain some of the nutritional programming effects of colostrum on neonate development.

Quality and quantity of diet impact growth performance, health, and reproductive outcomes [15,16]. Therefore, performance of gilts in response to differences in levels of colostrum intake may be driven by differences in nutrient availability (defined as dose of diet herein), level of bioactive factors (defined as diet type herein), or the interaction of diet and dose effects. We hypothesize that colostrum’s role in the metabolic transition of the neonate to the extra-uterine environment is primarily through its activation of lipid metabolic pathways in the first 24 h. To better understand the role of colostrum in the metabolic transition of neonates, we analyzed changes in circulating lipid and metabolite profiles and growth performance metrics at 24 h postnatal in response to colostrum. In particular, we aimed to determine the role that level of nutrients and bioactive factors in colostrum promote 24 h weight gain, immunocrit ratio, circulating levels of proteins, glucose, β-hydroxybutyrate (BHB), acetate, free amino acids, and lipid and small metabolite profiles. We asked the effects of standardized high and low doses of colostrum and milk replacer (MR) on these variables. We based the high (20% of birth body weight (BBW); COL20) and low colostrum dose (10% of BBW, COL10) on findings from a commercial study that reported a 60% mortality rate of piglets that ingested less than 100 g of colostrum during the first 24 h versus a 10% mortality rate of piglets ingesting more than 200 g [17], as well as our tests of standardized doses of homogenate colostrum samples [18]. In our previous study [18], the COL20 treatment group gained 15 times more weight than COL10 piglets in the first 24 h postnatal, had higher rectal temperature, and greater serum immunocrit ratios. After 24 h of COL feeding, both treatments were returned to their respective litters to nurse from sows. Despite similar average daily gains after return to sow, the growth trajectory differences between the treatments remained across the first week postnatal, consistent with findings from the study in a commercial setting [17]. In addition to the response to variable colostrum levels, we asked the effect of a relatively high level and low level of nutrient intake by bottle feeding piglets MR at 20% (MR20) or 10% (MR10) of BBW. A group of piglets that stayed on the sow (SOS) to suckle colostrum served as a positive control with the assumption of adequate intake. A sixth group of piglets was euthanized immediately after birth without being fed (zero hour, ZH) to serve as a negative control to express changes relative to those at birth. All other animals were euthanized after a 2 h fast following the final feeding to standardize piglets across treatments. To help in interpreting the response to colostrum intake on variables, data were interpreted within the context of comprehensive analysis of colostrum and MR diet, specifically free amino acid content, lipid profiles, metabolites, proteome, and macronutrient composition.

## Materials and methods

### Ethics statement and power analysis

The study protocol was reviewed and approved by Purdue University Animal Care and Use Committee (Protocol No. 2110002200) prior to beginning animal experiments. Pre-experimental power analysis using an alpha error of 0.05 indicated a treatment group size of 10 was adequate to achieve 0.99 statistical power if a 1.5-fold (50%) difference and standard deviation of 0.25-fold of any variable was identified.

### Animals and study design

**Fig 1:**
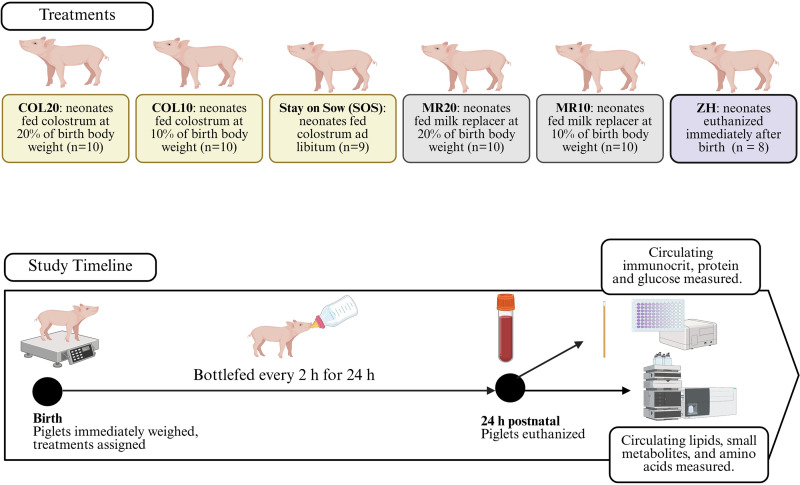
Experimental design of piglets fed colostrum (COL) at 20% (COL20) or 10% (COL10) of birth body weight, milk replacer (MR) fed at 20% (MR20) or 10% (MR10) of birth body weight, *ad libitum* intake of COL by stay-on-sow (SOS), or no feeding and immediately euthanized (ZH). At birth, piglets were not allowed to suckle, were immediately weighed, and allocated to treatments. Piglets on the COL20, COL10, MR20, and MR10 were fed every 2 hours from the first 24 h postnatal and piglets on the SOS were immediately returned to the sow for unlimited suckling. All piglets that received dietary treatments were euthanized at 24 h postnatal, blood was collected, and analyzed for glucose, protein, immunocrit, lipids, and small metabolites.

The study was conducted in two replicates that took place in August 2022 and October 2022 at the Animal Sciences Research and Education Center Swine Farm at Purdue University. Across both replicates, 57 gilt piglets were born to Landrace x Yorkshire sows terminally bred to Duroc sires. Piglets were born to 35 different sows with 18 sows used in the first replicate of the study and 17 in the second replicate. The average parity of the sows was 2.3 ± 1.43 (mean ± standard deviation). Farrowing was induced on day 115 of gestation using 2 mL of Lutalyse (Zoetis, Parsippany-Troy Hills, NJ, USA). Immediately after birth, piglets were prevented from suckling, towel-dried, and weighed. Piglets weighing 1.2 to 1.8 kg were selected for the study to minimize variation related to birth weight. Mean piglet BBW across all treatments ranged from 1.41 to 1.45 kg. Piglets were then randomly allocated to one of six treatments: 1) COL bottle-fed every 2 h at 20% of BBW (COL20; n = 10); 2) COL bottle-fed every 2 h at 10% of BBW (COL10; n = 10); 3); Ralco MR bottle-fed every 2 h at 20% BBW (MR20; n = 10); 4) Ralco MR bottle-fed every 2 h at 10% BBW (MR10; n = 10); 5) *ad libitum* colostrum intake by staying on the sow (SOS; n = 9); and 6) no COL or MR and immediately euthanized at birth to serve as the negative control (zero hour, ZH; n = 8; Fig 1). Treatments were assigned evenly across litters, and piglets from the same sow were not allocated to the same treatment. However, due to limitation of available gilts within the selected weight range, not all treatments were represented within a single litter.

The SOS group was returned to the sow to allow for *ad libitum* suckling. Gilts allocated to COL20, COL10, MR20, or MR10 treatments were moved from the farrowing house to a nursery in a separate building equipped with 50-cm × 40-cm pens, with slotted flooring and a heat lamp (Producer’s Pride, Tractor Supply) to prevent hypothermia. Twelve equal bottle feedings of a homogenate, pooled COL or MR were fed to gilts every 2 h over 24 h to reach a total intake of 20% or 10% of BBW. To determine the amount bottle fed to each piglet, the BBW (kg) was multiplied by either 0.20 (COL20 or MR20) or 0.10 (COL10 or MR10), then divided by 12 to calculate the amount of COL or MR fed every 2 h. Piglets were bottle-fed their respective treatments using Evenflo Feeding baby bottles (Evenflo, West Chester, OH). Bottles were cleaned using hot water and dish washing liquid between each feeding. If a piglet refused to eat, they were either gavaged or syringe-fed their dietary allocation.

Once piglets consumed their respective diets for 24 h, including SOS treatment, gilts were fasted for 2 h prior to scheduled euthanasia. Rectal temperature and weight were collected right before euthanasia. Piglets were euthanized via CO_2_ inhalation using gradual fill method (American Veterinary Medical Association), with an 80–90% CO_2_ concentration maintained for 5 minutes and a displacement rate of 10–30% of chamber volume/min. CO_2_ flow continued for 5 minutes after respiratory arrest. Immediately after euthanasia, a blood sample was collected from the jugular vein into a 10 mL serum separator tube (BD Vacutainer, Franklin Lakes, NJ). The blood was allowed to clot, and then centrifuged at 10,000 × g for 10 min to separate serum, which was aliquoted into multiple microcentrifuge tubes and stored at −80°C until further analysis.

### Diets

Pooled COL fed to the COL20 and COL10 pigs was collected over a two-week period from a commercial swine farm in Indiana. Approximately 50–100 mL of COL was collected from ~100 sows during active farrowing. Colostrum was pooled to obtain the ~ 9 L needed to feed the COL20 and COL10 piglets. An aliquot of the pooled COL was sent to the Animal Disease Diagnostic Lab at Purdue University (West Lafayette, IN) to test for porcine circovirus and porcine reproductive and respiratory syndrome, and all sample results came back negative. Colostrum was frozen in two separate pools, one for the first study replicate and one for the second study replicate. Prior to the beginning of each study replicate, COL was thawed at 4°C for 72 h and kept at 4°C throughout the study.

Ralco Birthright baby pig MR (Ralco Show, Marshall, MN) was mixed following the manufacturer’s instructions for orphaned piglets, dissolving 500 g in 3.78 L of tap water. Ralco MR ingredient and nutrient composition are presented in Supplemental Table S1 [19]. Fresh MR powder was mixed for each study replicate. The homogenate MR was stored at 4°C throughout the study and the MR container was inverted to mix prior to dispensing for the 2 h feedings. Bottles with dispensed diets for both COL and MR were placed in a 37°C water bath for 10 min to warm prior to feeding. Diet samples were collected from the pooled COL and MR and stored at −80°C until further analysis. Colostrum from the sows (n = 5) that the SOS gilts suckled from was collected manually approximately 3 h after the initiation of farrowing and stored at −80°C until further analysis.

### Analysis of dietary energy and nitrogen

Fifteen grams of pooled COL or COL from SOS sows was weighed, freeze-dried in a FreezeZone Bulk Tray Dryer (Labconco, Kansas City, MO) for 24 h, and a dry weight was collected to calculate the dry matter of the diet. Five grams of the dried MR powder was used for nitrogen and gross energy analysis. The dried samples were analyzed in triplicate for nitrogen using Leco TrueMac Nitrogen Analyzer (Leco Corporation, St. Joseph, MI). The nitrogen values were then multiplied by 6.38, the coefficient for milk, to obtain crude protein values on a dry matter basis. Gross energy (% dry matter) was measured using Parr 6200 bomb calorimeter (Preiser Scientific, Louisville, KY) equipped with a Parr 6510 Water Handling System.

### Analysis of dietary triglycerides and protein and serum immunocrit, glucose, and protein

Dietary triglyceride content (as-fed basis) was analyzed using a Fujifilm Triglyceride LabAssay (Fujifilm, Lexington, MA; detection range 100–888 mg/100mL), samples were diluted 1:10 in nanopure water, and analyzed in triplicate on the same plate. Serum and dietary protein (as-fed basis) were analyzed using a Pierce™ BCA Protein Assay (ThermoFisher Scientific, Waltham, MA; detection range 20–2000 μg/mL), and both sample types were diluted 1:100 in 1X phosphate buffered saline (PBS). Diet samples for protein analysis were analyzed on the same plate in triplicate, but serum samples were analyzed in triplicate on three separate plates due to the number of samples. Plates were balanced by treatment and study replicate, and the inter-assay coefficient of variation (CV) for serum protein was 2.76%. Serum glucose was analyzed using Fujifilm Glucose Autokit (Fujifilm, Lexington, MA; detection range up to 700 mg/dL), samples were diluted 1:5 in nanopure water prior to analysis, and analyzed in triplicate on three separate plates. Plates were balanced by treatment and study replicate. The inter-assay CV for serum glucose was 2.10%. Serum immunocrit ratio was measured following the protocol by [20]. For each assay, the average of triplicate was used for calculation of concentration for statistical analysis. One sample of pooled COL and one sample of MR was analyzed per study replicate (n = 2 per diet type). All COL samples from SOS sows were individually measured with the total number of samples across replicates being 5. Attempts to measure glucose in diets and triglycerides in serum samples found that levels were below the detectable limits of the commercial assays and were therefore not included in the analysis.

### Liquid chromatography tandem mass spectrometry (LC-MS/MS) analysis of dietary lactose content and free amino acids in piglet serum and diet

Lactose content of pooled COL, COL from SOS sows, and MR was analyzed in the skim milk fraction of diet samples diluted 1:10 in nanopure water. Diluted samples were spiked with 10 µL of 1 mg/mL of [U-^13^C] lactose. The spiked sample was combined with 100 µL of acetonitrile, vortexed for 5 min, centrifuged at 13,000 x g for 8 min, and the supernatant transferred to a high performance liquid chromatography (HPLC) vial and analyzed using liquid chromatography tandem mass spectrometry (LC-MS/MS) as described in [21]. The concentration of lactose in samples was determined in relation to spectra of the [U-^13^C] lactose standard. The lactose content of diets was analyzed in two separate experiments to confirm values and values were consistent between experiments. Data from the initial analysis is presented.

Samples for analysis of free amino acids in serum and diets were prepared as previously described in [21] with some modifications. Thirty microliters of serum or diet sample was brought to 100 µL using 1X PBS and spiked with 5 µL of 100 ng/µL of d5-glutamic acid (500 ng final concentration) internal standard. Samples were combined with 100% trichloroacetic acid, vortexed, and centrifuged at 13,000 x g for 8 min, after which, 50 µL of the supernatant was combined with 50 µL of 100% acetonitrile into a HPLC vial. Samples were analyzed with LC/MS-MS using parameters outlined in [21]. Concentrations of amino acids were determined in relation to spectra of the d5-glutamic acid internal standard. To determine alterations in concentration of amino acids in diets, the fold difference (COL/MR) and Log2 fold change were calculated.

### Liquid chromatography mass spectrometry analysis of serum β-hydroxybutyrate and acetate

Serum samples were analyzed for BHB and acetate using multiple reaction monitoring (MRM). Samples were extracted by combining 100 μL of serum with 10 μL of a 2 mg/10 μL in water solution of N-(3-Dimethylaminpropyl)-N’ethylcarbodiimide hydrochloride and 10 μL of a 6 M aniline solution diluted in 50% HCl. Samples reacted for 2 hours with gentle shaking at 400 rpm at room temperature (RT). The reaction was quenched using 3 μL of triethylamine. Samples were immediately vortexed, and centrifuged at 13,000 rpm for 5 min at RT. Samples were combined with an equal volume of working standard solution (3.3 ng/μL of BHB, 3.3 ng/μL acetate, and 6 M ^13^C_6_ aniline) in a HPLC vial. An Agilent 1200 Rapid Resolution liquid chromatography (LC) system coupled to an Agilent 6460 series QQQ mass spectrometer (MS) was used to analyze BHB and acetate in each sample. A Waters Atlantis T3 (2.1mmx75mm, 3um) column (Waters Corporation Milford, MA) was used for LC separation. HPLC grade water with 0.1% formic acid (v/v) was used as mobile phase A. HPLC grade acetonitrile with 0.1% formic acid (v/v) was used as mobile phase B. The linear LC gradient was as follows: time 0 min, 10% B; time 0.5 min, 10% B; time 12 min, 40% B; time 13 min, 98% B; time 14 min, 10% B; time 20 min, 10% B. The flow rate was 0.4 mL/min. The data were acquired in positive electrospray ionization (ESI) mode. The jet stream ESI interface had a gas temperature of 325°C, gas flow rate of 10 L/min, nebulizer pressure of 40 psi, sheath gas temperature of 250°C, sheath gas flow rate of 7 L/min, capillary voltage of 4000V in positive mode, and nozzle voltage of 1500 V. The Δ electron multiplier voltage was 400. The concentration of BHB and acetate were determined from the relative response between the aniline labeled sample verses ^13^C_6_ aniline labeled standard. All data were analyzed with Agilent Masshunter Quantitative Analysis (Version 10.1).

### Liquid chromatography tandem mass spectrometry (LC-MS/MS) analysis of dietary global proteome

Protein isolation and LC-MS/MS analysis were performed in the Proteomics Core of Bindley Bioscience Center at Purdue University. For LC-MS/MS proteome analysis three MR and three pooled COL diet samples were analyzed as technical replicates. Protein was isolated and shotgun label-free LC-MS/MS was conducted as we previously described for sow milk proteome analysis [22]. MaxQuant software (version 1.6.3.3) was used to map LC-MS/MS data to proteins in the *Sus scrofa* sequence database for the COL diet and *Bos taurus* sequence database for the MR diet, both of which were downloaded from UniProt (www.uniprot.org). The parameters used to map LC-MS/MS data included precursor mass tolerance of 10 ppm, enzyme specificity of trypsin/Lys-C allowing up to 2 missed cleavages, oxidation of methionine (M) and Acetyl N-term as variable modifications and iodoethanol (C) as a fixed modification. False discovery rate (FDR) of peptide spectral match and protein identification was set to 0.01. Results were filtered to retain only proteins with label-free quantification (LFQ) > 0 and MS/MS (spectral counts) ≥ 2 for further analysis.

To compare the distribution of proteins within and across diets the relative distribution of a protein within a diet was determined. Briefly, for each protein measured in a diet the average LFQ intensity was calculated across replicate diet samples, and then the average LFQ of all proteins measured was summed and used as the denominator to calculate the relative distribution of proteins. The relative distribution was calculated by dividing individual LFQ by the sum of the intensities within diet type. If Uniprot ID mapped to the same protein, duplicate values were summed. All immunoglobulin and immunoglobulin like proteins, including J-chain, were considered duplicate proteins and intensities were summed to give one value for the group of immunoglobulin proteins.

### Analysis of lipidome of serum using MRM profiling

Lipid extraction was performed according to the Bligh & Dyer protocol [23]. Briefly, 150 μL of ultrapure H_2_O was added to a microtube containing 50 μL of serum or diet. Then, 550 μL of methanol (MeOH) and 250 μL of chloroform (CHCl_3_) + 0.01% butylated hydroxytoluene) were added and vortexed for 10 seconds, then the samples were incubated for 15 min at 4 °C. Next, another 250 μL of ultrapure H_2_O and 250 μL of CHCl_3_ were added. Samples were then centrifuged at 15,000 × g for 10 min to enhance polar from nonpolar phase separation (two-phase solution). The bottom, organic phase containing lipids was transferred to a new tube. All lipid extracts from samples were dried in a Speedvac (Savant SpeedVac AES2010, ThermoFisher Scientific, San Jose, CA). Dried lipid extracts were stored at −80°C until mass spectrometry analysis.

### Lipidome analysis discovery phase evaluation of serum and diet samples

Lipidome analysis using MRM profiling is conducted in two phases: the discovery and screening phase. Six serum samples pooled by treatment and individual diet extracts of pooled COL, MR, and COL collected from SOS sows were analyzed during the discovery phase. Samples were profiled for a list of MRM corresponding to lipid species information, which includes lipid class as well as total carbon length and number of unsaturated bonds across fatty acyl groups, using the expected mass charge (m/z) ratio of the parent and product ion related to a phospholipid polar head or a fatty acid neutral loss acquired from the LipidMAPS online database [24–26]; http://www.lipidmaps.org/). Constitutional isomers were combined into one single MRM. This strategy allowed compaction of the large number of entries (16,257) by one order of magnitude (3,437 MRMs in the final discovery phase list). The 3,437 MRMs included in the discovery phase were used to interrogate a pooled serum sample or individual diet sample. Due to the limited time of signal at flow injection, the 3,437 MRM were organized into 27 separate lists of MRM; each one involved 2 min of data acquisition per flow injection, and each list of MRM included a maximum of 500 MRM transitions. The lipid extracts of the diets or pooled serum samples were diluted in ACN + MeOH + 300 mM ammonium acetate (NH_4_Ac) 3:6.65:0.35 (v/v) and flow-injected (8 μL) to the ESI source of an Agilent 6410 QQQ liquid chromatography-mass spectrometer (Agilent Technologies, Santa Clara, CA, USA) using a micro-autosampler (G1377A). The precursor ion selection window was 0.7 Thomson. The capillary pump connected to the autosampler operated with a flow of 20 μL/min and a pressure of 150 bar. Capillary voltage on the instrument was 3.5–5 kilovolts and the gas flow was 5.1 L/min at 300°C.

### Lipidome analysis screening phase evaluation of serum samples

The screening phase is the second phase of MRM profiling and performed on individual samples. The 769 MRM classified as detected in the pooled samples during the discovery phase were used for the screening phase. For an MRM to be classified as detected, its raw ion signal intensity values were 1.3-fold greater than the blank. Individual samples were prepared for the screening phase using the same steps as above but were spiked with EquiSplash LIPIDOMIX Quantitative Mass Spec Internal Standard (Avanti Research, Alabaster, AL, USA). MRM with ion intensities 1.3-fold greater than the blank in the screening phase were selected for downstream analysis.

### Lipidome data normalization by internal standard and sum of ion intensity values within lipid class

MRM data were normalized by two approaches: 1) internal standard (IS) for semi-quantitative analysis and 2) sum of ion intensities to assess relative distribution patterns. Data normalized by IS were used to calculate concentration (arbitrary units) of triacylglycerol (TG), diacylglycerol (DG), and cholesteryl esters (CE). The other lipid classes: phosphatidylcholine (PC), phosphatidylethanolamine (PE), phosphatidylinositol (PI), phosphatidylserine (PS), phosphatidylglycerol (PG), sphingomyelin (SM), and ceramide (Cer) lipid classes were defined as phospholipid (PL) + Cer lipids. These lipids were normalized by their respective IS; then, the normalized values of PC, PE, PI, PS, PG and SM, were summed to obtain a total concentration of PL. The ratio of TG + CE/PL + Cer was calculated as an indirect measure of relative size of lipoproteins or chylomicrons in circulation because the core of these molecules is comprised of TG and CE and is distinct from the lipids that encompass the PL membrane. Carnitine esters and free fatty acids included in MRM screening phase do not have an internal standard, and as such, were not normalized by IS.

Data were also normalized by sum of the ion intensities within lipid class (i.e., normalization by sum) to analyze change in distribution of lipids in response to treatments. Following normalization by sum, data were uploaded into MetaboAnalyst 6.0 [27] for statistical analysis. Variance filter was reduced to 0 and data were autoscaled to ensure normal distribution. Principal component analysis (PCA) and hierarchical clustering (including heatmaps with dendrograms) using the Ward method were conducted. One-factor analysis was applied using volcano plot for biological questions on impact of adequate COL (COL20 and SOS vs ZH) and impact of adequate MR (MR20 vs ZH) on lipid distribution.

To examine the impact of diet (COL vs MR) and dose (20% vs 10%) on lipid distribution, two independent linear models were constructed using the independent PCA function within Metaboanalyst 6.0. The first model, the diet model, used COL vs MR as the primary experimental factor, whereas the dose of food (20% and 10%) was a covariate. For the second linear model, the dose of food (20% vs 10%) was used as the primary experimental factor whereas diet (COL and MR) was used as a covariate. Data generated from these analyses included log base 2-fold (log2FC) differences between doses and COL versus MR, and an FDR < 0.05 was used as the cut-off to identify MRM different between groups. To understand how factors affected the distribution of lipids by carbon length and unsaturated bond number, statistically different lipids within TG and PL + Cer (SM, PC, PG, PI, PS, PE, and Cer) were grouped, and the percent increased or decreased within carbon chain length or unsaturated bond number category were calculated.

### Analysis of lipidome of diet using MRM profiling

MRM-profiling of lipids in COL and MR samples from first and second study replicates along with the 5 colostrum samples from the sows that suckled the SOS piglets was conducted. Data were normalized using IS to calculate the concentration (arbitrary units; AU) of lipid classes in the diets. MRM data normalized by sum were used to analyze differences in distribution of lipids within a class. Note, diet lipidome data are descriptive. To meet the minimum input of n = 3 needed for univariate analysis in Metaboanalyst 6.0, a composite value was generated by averaging values from COL and MR samples from the first and second study replicates and used as a ‘dummy’ sample. This pseudo-replicate approach was used only for visual representation and not for referential statistics.

### Small metabolite analysis in serum and diet

MRM-profiling of small metabolites of serum and diet samples was also conducted. During the phase separation step of lipid extraction of samples, lipids separate into the bottom, organic phase, whereas the small metabolites will go to the top, aqueous phase. For analysis of small metabolites, the top aqueous phase was removed and dried in a Speedvac (Savant SpeedVac AES2010, ThermoFisher Scientific, San Jose, CA). Samples were stored at −80°C until analysis. Samples were reconstituted in 50% acetonitrile in water, then diluted in ACN + MeOH + 300 mM NH_4_Ac 3:6.65:0.35 (v/v) in an HPLC vial. Samples were analyzed using the same LC-MS/MS instrument and parameters as above. Small metabolite analysis was performed by flow injection and all attributions are tentative. Data were normalized by the sum of the ion intensities. Small metabolites were evaluated using two methods: one positive ion and one negative ion method. There were 326 metabolites evaluated using the negative ion method and 371 in the positive ion method, with 67 and 156 serum metabolites 1.3-fold greater than the blank in each method, respectively.

Normalized serum small metabolite data were uploaded into MetaboAnalyst 6.0 for statistical analysis and metabolites identified as different between treatment groups were uploaded into MetaboAnalyst 6.0 for enrichment analysis. Upon autoscaling for normal distribution, one-factor analysis was applied using volcano plot to ask the question if the impact of adequate COL (SOS and COL20) or adequate MR (MR20) impacted circulating small metabolite data compared to ZH piglets. The next question utilized a linear model to ask if diet (COL vs MR) or dose (20% vs 10%) impacted circulating small metabolite levels. All diet and serum amino acid, proteome, lipidome, and small metabolite diet were uploaded to Purdue University Research Repository. These data presented as Supplemental Tables and Figures can be found using https://doi.org/10.4231/KN6Y-7S80 [19].

### Statistical analysis

The study was designed to ask two questions: 1) what was the effect of treatment (SOS, COL20, COL10, MR20, MR10, ZH) on variables of interest, including serum protein, immunocrit, glucose, BHB, acetate, amino acids, and lipid concentration, and 2) what was the effect of diet (COL20, COL10 versus MR20, MR10) and dose of diet (COL20, MR20 versus COL10, MR10) on the variables of interest? To test the effect of treatment, data were analyzed using the PROC MIXED procedure of SAS 9.4 (SAS, Cary, NC) following a check for normality using PROC Univariate procedure with the Shapiro Wilk test *P*-value evaluated given the strength of the Shapiro Wilk compared to Kolmogorov–Smirnov and that the sample size was close to 50. The following model was used:


$${\text{Y}}_{\text{ij}}=\mu +{\text{a}}_{\text{i}}+{\text{b}}_{\text{j}}+{\text{e}}_{\text{ij}}$$

Y_ij_ is the response variable, µ is the overall mean; a_i_ is the fixed effect of i^th^ treatment, b_j_ is the fixed effect of the j^th^ study replicate, and e_ij_ is the residual. Outliers were evaluated using the “influence” command in SAS to generate Cook’s D values. Data points with a Cook’s D value above 10/n were considered an outlier and removed [28]. Tukey’s honest significant difference post hoc analysis was used to separate means.

To evaluate if diet (COL20, COL10 versus MR20, MR10) and dose of diets (COL20, MR20 versus COL10, MR10) impacted dependent variables, a separate model with the individual effects of diet, dose, and the interaction of diet and dose was used. To utilize this model, the SOS and ZH treatments were removed from analysis. The following model was used:


$${\text{Y}}_{\text{ijk}}=\mu +{\text{a}}_{\text{i}}+{\text{b}}_{\text{j}}+{\text{ab}}_{\text{ij}}+{\text{c}}_{\text{k}}+{\text{e}}_{\text{ijk}}$$

Y_ijk_ is the response variable, µ is the overall mean; a_i_ is the fixed effect of i^th^ diet, b_j_ is the fixed effect of the j^th^ dose, ab_ij_ is the fixed effect of the interaction of the i^th^ diet and j^th^ dose, c_k_ is the fixed effect of the k^th^ study replicate, and e_ijk_ is the residual. Outliers were evaluated using the “influence” command in SAS to generate Cook’s D values [28]. Data from both analyses were considered significant at *P* ≤ 0.05 and a tendency was determined at 0.05 < *P* ≤ 0.10. Diet proteome, lipidome, and metabolite profile were analyzed in Metaboanalyst 6.0 to determine differentially abundant proteins, lipids, and metabolites in the COL, COL from SOS sows, and MR diets. Dietary macronutrient and amino acid composition were not statistically analyzed.

## Results

### Validation of experiment design through evaluation of rectal temperature and body weight change in the first study replicate

In addition to being part of the overall study, the first experimental replicate aimed to test whether the MR20 and MR10 groups had similar responses in change in body weight and rectal temperature at 24 h postnatal to COL20 and COL10 groups. The ability to maintain rectal temperature is indicative of adequate intake to thermoregulate. A total of 18 gilts were assigned to 1 of the 6 treatments in the first study replicate, as described in Materials and Methods (n = 3 per treatment group). Analysis of variance indicated that treatment significantly impacted rectal temperature at 24 h (*P* < 0.05). Tukey’s separation of means found that rectal temperature (mean ºC ± standard deviation) was similar (*P* > 0.05) between MR20 (39.2ºC ± 0.4) and COL20 (39.1ºC ± 0.3). Rectal temperature was also similar between COL10 (37.5ºC ± 2.1) and MR10 (37.6ºC ± 0.7), but differed between 20% (COL20 and MR20) and 10% (COL10 and MR10) groups (*P* < 0.05). Analysis of 24 h weight change (mean kg ± standard deviation) identified animals in the 20% groups (COL20 0.10 ± 0.04 kg and MR20 0.03 ± 0.04 kg) gained weight during the 24 h bottle feeding, and COL20 and MR20 were not different from one another (*P* > 0.05). However, on average, COL10 (−0.08 ± 0.04 kg) and MR10 (−0.003 ± 0.04 kg) lost weight during this period, and also were not different from one another *(P* > 0.05). The finding that animals in the MR20 and COL20 groups in the first replicate had similar responses to their respective diets indicates that diet type (COL or MR) does not limit the animals’ ability to maintain rectal temperature and body weight gain. However, dose of diet (20% vs 10%) appears to limit rate of gain and maintenance of rectal temperature as demonstrated by reductions in both of these variables for the MR10 and COL10 treatments. Ultimately, these preliminary responses from the first study replicate supported the study design.

### Analysis of diet composition

The primary ingredients of the MR included dried whey, dried skim milk, casein, dried whey protein concentrate, porcine plasma, animal fat, and coconut oil (Supplemental Table S1 [19]). Analysis of the gross energy content of the diets found pooled COL to have double the kcal in 200 mL dose of COL per kg of animal compared to MR (Table 1). It appears most of the difference in gross energy is due to a higher solid content (i.e., higher dry matter content of COL). The level of protein in pooled COL was higher and likely the main nutrient contributor to gross energy as there were less lactose and triglycerides in COL than MR.

**Table 1:** Nutrient composition of pooled colostrum collected from a commercial swine farm and commercial swine milk replacer fed to piglets every 2 hours for 24 hours at either 20% or 10% of their birth body weight.

Nutrient Composition	Pooled Colostrum^1^		Milk Replacer^2^			
	Amount	kcal/200 mL/kg of animal	Amount	kcal/200 mL/kg of animal		
Dry Matter (DM), %	22.1	44.2	13.2	26.4		
Nitrogen content, % of DM	9.40	18.8	3.60	7.20		
Crude Protein, % of DM	60.1	120	23.2	46.4		
Gross Energy, kcal/g of DM	5.87	260	4.76	126		
Nutrient Composition	**Pooled Colostrum** ^**1**^			**Milk Replacer** ^**2**^		
	Amount	g in 200 mL/kg of animal	kcal/200 mL/kg of animal	Amount	g in 200 mL/kg of animal	kcal/200 mL/kg of animal
Protein, g/mL as-fed^3^	0.12	24.0	96.0	0.06	12.0	48.0
Lactose, g/mL as-fed^3^	0.03	6.00	24.0	0.12	24.0	96.0
Triglycerides, g/mL as-fed^3^	0.025	5.00	45.0	0.03	6.00	54.0

^1^Colostrum collected from ~100 commercial sows in 50–100 mL aliquots and pooled together.

^2^Ralco commercial swine milk replacer.

^3^Analysis of nutrient composition was conducted on an as-fed basis

Analysis of diets for free amino acid (Supplemental Table S2 [19]), proteome (Supplemental Table S3 and S4 [19]), lipid profiles (Supplemental Table S5, S6, S7, S8 [19]), and small metabolites (Supplemental Table S9 and S10 [19]) found they also varied on an as-fed basis. The concentration of four amino acids: serine (Ser), asparagine, leucine (Leu), and glutamine (Gln), was higher in pooled COL (0.90, 0.20, 1.52 and 1.61 ng/µl) than MR (0.15, 0, 1.08, and 1.22 ng/µl, respectively; Supplemental Table S2 [19]). The concentration of the other 16 amino acids measured were all greater in MR than pooled COL, with tyrosine, proline, histidine, lysine (Lys), glutamate (Glu), and methionine (Met) much higher in MR (6.0, 62.25, 1.73, 4.37, 91.33, 688 ng/µl, respectively) than pooled COL (1.6, 15.74, 0.48, 0.51, 8.69, 0.2 ng/µl, respectively). Lysine and Met were supplemented into MR by the manufacturer, and the source of the other amino acids was from dried whey, casein and skim milk (Supplemental Table S1 [19]).

Of the 558 and 583 unique (non-duplicate) proteins measured in MR and pooled COL diets, 272 overlapped. Immunoglobulin proteins were the most abundant in both diets. The relative distribution of the remaining 271 overlapping proteins differed between diets (Supplemental Table S4 [19]).

Analysis of lipid profiles of diets indicated that the concentration (AU, Supplemental Table S8 [19]) of TG was similar between COL (359.67 AU) and MR (321.78 AU) on an as-fed basis. However, DG concentration was higher in MR (257.04 AU) than COL (34.24 AU). The level of PI and PC lipids was higher in MR (4.59 and 18.37 AU) than COL (2.66 and 11.34 AU), whereas PE was less in MR (3.68 A) than COL (5.79 AU). Other lipid classes did not differ between COL, MR, and SOS treatments.

Multivariate analysis of the relative distribution of lipids in diets indicated large differences in lipid profiles between diets fed to COL and MR gilts, whereas dietary lipids of COL and SOS treatments were more similar (Supplemental Fig S1 [19]). To better understand differences in dietary lipids, the distribution of lipids by carbon length and number of unsaturated bonds across the fatty acyl groups in the TG and PL + Cer (PC, PE, PG, PI, PS, SM, Cer) were graphed (Supplemental Fig S2 and S3 [19]). Milk replacer had a greater distribution of TG ≤ 46 carbons, whereas pooled COL had a greater enrichment of TG ranging from 49 to 52 and ≥ 59 total carbons across the three fatty acyl groups. Milk replacer had a greater distribution of TG with ≤ 2 unsaturated bonds than COL, and COL had a greater distribution of TG with 4–6 and 10–13 unsaturated bonds across the fatty acyl groups. Milk replacer had a greater distribution of TG with 14:0, 16:0, 18:0, and 20:0 fatty acids, and COL had a greater distribution of TG with 16:1, 18:2, 18:3 and 20:4. Milk replacer had a greater distribution of PL + Cer with 21–32 carbons, whereas COL was more enriched with PL + Cer 41–44 carbons across the fatty acyl chains. Phospholipids + Cer in MR had fewer unsaturated bonds in the fatty acyl chains than pooled COL with 0–1 unsaturated bonds in MR compared to COL. Colostrum had a greater distribution of PL + Cer with 4 or more unsaturated bonds across the two fatty acyl groups.

Exploratory analysis of metabolites using MRM-profiling also indicated differences in distribution between the diets (Supplemental Table S10 [19]). Colostrum had greater levels of uridine, taurine, myo-inositol, inosine, guanosine, D-biotin than MR. Levels of maltose, D-fructose, sucrose, lactose, and lactic acid were proportionally greater in MR than pooled COL.

### Impact of treatment, diet type, and dose of diet on piglet weight change and rectal temperature during the first 24 h postnatal

**Fig 2:**
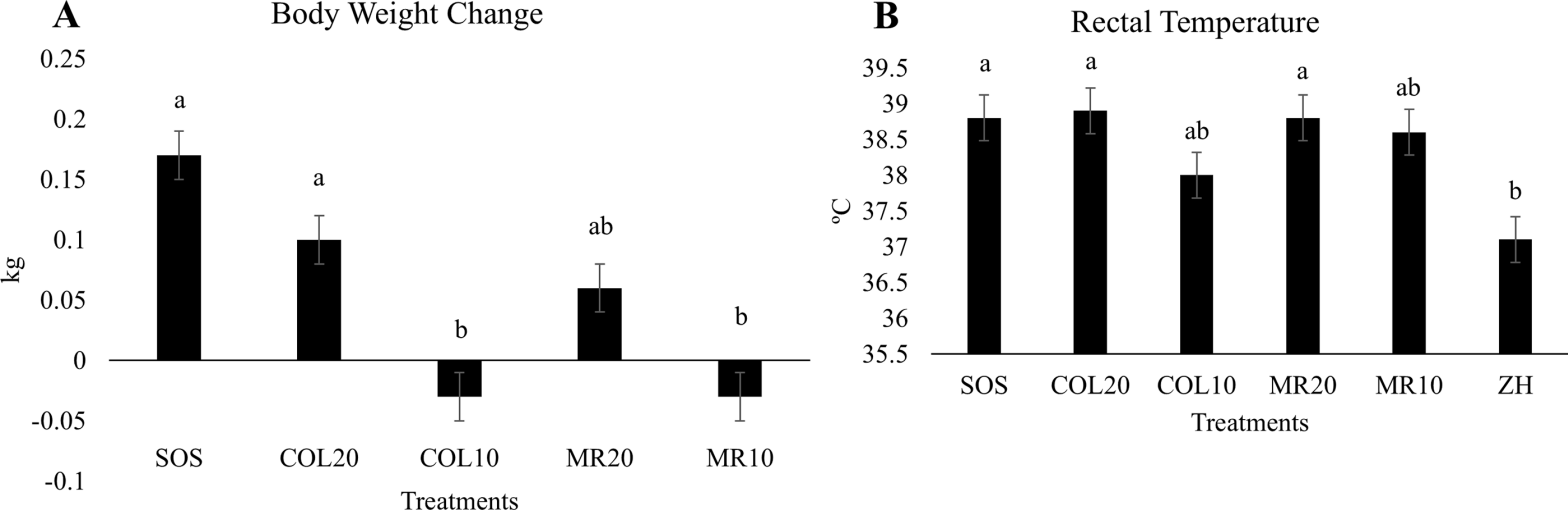
Impact of colostrum (COL) fed at 20% (COL20) or 10% (COL10) of birth body weight, milk replacer (MR) fed at 20% (MR20) or 10% (MR10) of birth body weight, *ad libitum* intake of COL by stay-on-sow (SOS), or no feeding and immediately euthanized [zero hour (ZH)] on A) body weight change over 24 h and B) rectal temperature at 24 h. Separation of means was generated by Tukey’s honest significant difference, and bars without a common letter differ (*P* ≤ 0.05).

Consistent with the study design, all treatments had similar mean BBW (Table 2). Analysis of the change in weight between birth and 24 h postnatal, found treatment significantly impacted 24 h weight gain (*P* < 0.0001). Piglets on the SOS [least squares means (lsmeans) ± standard error of the mean (SEM); 0.17 ± 0.02 kg], COL20 (0.10 ± 0.02 kg), and MR20 (0.06 ± 0.02 kg) treatments gained a similar amount of weight over the 24 h period of feeding (*P* > 0.05), whereas piglets on the COL10 (−0.03 ± 0.02 kg) and MR10 (−0.03 ± 0.02 kg) treatments lost weight, demonstrating an overall dose effect on weight gain (Fig 2A and Table 2; dose *P* < 0.0001). Piglets on the SOS (lsmeans ± SEM; 38.8 ± 0.32°C), COL20 (38.9 ± 0.32°C), or MR20 (38.8 ± 0.32°C) treatments had a rectal temperature within normal range for a neonatal piglet (normal range: 38.6°C to 39.2°C; Fig 2B and Table 2), and were not different from one another (*P* > 0.05). Mean rectal temperature of MR10 was at the low end of normal (38.6 ± 0.32°C) and the COL10 group had a mean rectal temperature that was below normal (mean = 38.0°C), although neither of these treatments differed significantly from SOS, COL20 and MR20 piglets (*P* > 0.05). The ZH piglets were considered hypothermic at 37.1°C, and differed from SOS (Tukey *P* = 0.003), COL20 (Tukey *P* = 0.01), and MR20 (Tukey *P* = 0.03), but not COL10 or MR10 (*P* > 0.05, Table 2, Fig 2B).

**Table 2:** Body weight at birth and 24 h, change in body weight at 24 h, rectal temperature at 24 h, and serum immunocrit, protein, glucose, β-hydroxybutyrate, and acetate responses to colostrum (COL) fed at 20% (COL20) or 10% (COL10) of birth body weight, milk replacer (MR) fed at 20% (MR20) or 10% (MR10) of birth body weight, piglets stayed on the sow and received *ad libitum* COL (SOS), and piglets did not receive food and were immediately euthanized after birth (zero hour; ZH). Two separate statistical models were used to evaluate the response variables to the effect of treatment, effect of diet (COL20, COL10 vs MR20, MR10), effect of dose of diet (COL20, MR20 vs COL10, MR10) or the interaction of diet and dose.

Variable	Lsmeans^1^						SEM^2^
	SOS	COL20	COL10	MR20	MR10	ZH	
Birth body weight, kg	1.42	1.44	1.44	1.45	1.41	1.43	0.04
Body weight at 24 h postnatal, kg	1.61	1.55	1.42	1.54	1.39	ND	0.06
Body weight change over 24 h, kg	0.17^a^	0.10^a^	−0.03^b^	0.06^ab^	−0.03^b^	ND	0.02
Rectal temperature, °C	38.8^a^	38.9^a^	38.0^ab^	38.8^a^	38.6^ab^	37.1^b^	0.32
Serum glucose, mg/dL	166.1^a^	128.9^ab^	93.8^b^	149.9^ab^	115.7^ab^	103.7^ab^	14.5
Serum protein, mg/mL	52.4^a^	44.4^a^	31.6^b^	15.2^c^	13.6^c^	17.4^c^	2.55
Serum immunocrit ratio	0.11^a^	0.11^a^	0.04^b^	0.03^bc^	0.02^c^	0.02^bc^	0.006
Serum β-hydroxybutyrate, ng/μL	2.23^bc^	3.23^a^	2.59^ab^	1.67^c^	1.75^c^	1.91^bc^	0.18
Serum acetate, ng/μL	1.49^ab^	1.30^ab^	1.24^b^	1.48^ab^	1.30^ab^	1.95^a^	0.15
**Variable**	***P*-values**						
	**Trt**	**Rep**	**Diet**	**Dose**	Diet * Dose	**Rep**	
Birth body weight, kg	0.99	0.37	0.85	0.66	0.62	0.21	
Body weight at 24 h postnatal, kg	0.04	0.47	0.76	0.03	0.87	0.41	
Body weight change over 24 h, kg	<0.0001	0.48	0.42	<0.0001	0.33	0.36	
Rectal temperature, °C	0.002	0.63	0.39	0.07	0.21	0.19	
Serum glucose, mg/dL	0.009	0.07	0.15	0.02	0.97	0.009	
Serum protein, mg/mL	<0.0001	0.34	<0.0001	0.02	0.04	0.62	
Serum immunocrit ratio	<0.0001	0.22	<0.0001	<0.0001	<0.0001	0.35	
Serum β-hydroxybutyrate, ng/μL	<0.0001	0.03	<0.0001	0.15	0.07	0.04	
Serum acetate, ng/μL	0.05	<0.0001	0.37	0.36	0.65	0.04	

^1^Least square means of independent variables in response to piglets fed different levels of COL or MR.

^2^Standard error of mean.

^(a-c)^Separation of means was generated by Tukey’s honest significant difference. Means without a common letter differ (*P* ≤ 0.05).

ND = not determined.

### Impact of treatment, diet, dose of diet, and interaction of diet and dose on circulating glucose, protein, and immunocrit

Treatment significantly impacted circulating levels of glucose (Trt *P* = 0.009), protein (Trt *P* < 0.0001), and immunocrit (Trt *P* < 0.0001). Piglets on the SOS treatment had the highest level of circulating glucose (lsmeans ± SEM; 166.1 ± 14.5 mg/dL), but this only differed from the COL10 piglets (93.8 ± 14.5 mg/dL), which had the lowest circulating glucose levels (Tukey *P* = 0.01). Although circulating glucose was not affected by diet (*P* = 0.15), dose had an overall effect (*P* = 0.02), with piglets consuming 20% of BBW (COL20 and MR20) having significantly higher levels of glucose (COL20 128.9 ± 14.5 mg/dL; MR20 149.9 ± 14.5 mg/dL) than those consuming 10% (COL10 93.8 ± 14.5 mg/dL mg/dL and MR10 115.7 ± 14.5 mg/dL; Table 2).

Circulating levels of protein varied by treatment with levels higher in SOS (52.4 ± 2.55 mg/mL), COL20 (44.4 ± 2.55 mg/mL), and COL10 (31.6 ± 2.55 mg/mL) than both MR (MR20 15.2 ± 2.55 mg/mL and MR10 13.6 ± 2.55 mg/mL) groups and ZH animals (17.4 ± 2.55 mg/mL; Trt *P* < 0.0001; Table 2). There were also diet (*P* < 0.0001), dose (*P* = 0.02), and diet by dose (*P* = 0.04) effects on circulating protein concentration with levels greater in COL (COL20, COL10) piglets than MR (MR20, MR10). COL20 was greater than COL10 (Tukey *P* = 0.009), but there was no difference in protein levels between MR20 and MR10 (*P* > 0.05). The significant interaction of diet by dose (*P* = 0.04) was driven by the COL20 treatment having the highest circulating levels of protein (Table 2).

Immunocrit is an indirect measure of immunoglobulins, and thus a way to evaluate passive transfer of immunity [20]. Compared to ZH (i.e., birth), immunocrit was significantly higher in SOS (lsmeans ± SEM; 0.11 ± 0.006; Tukey *P* < 0.0001) and COL20 piglets (0.11 ± 0.006; Tukey *P* < 0.0001), and tended to be slightly higher in COL10 (0.04 ± 0.006; Tukey *P* = 0.09). However, ZH was not different from MR20 (0.03 ± 0.006; Tukey *P* > 0.05) and MR10 piglets (0.02 ± 0.006; Tukey *P* > 0.05). Analysis of the overall effects of diet, dose, and diet by dose found immunocrit was significantly higher in COL (COL20 and COL10) than MR (MR20 and MR10, diet *P* < 0.0001) and higher in 20% (COL20 and MR20) than 10% (COL10 and MR10; dose *P* < 0.0001) with this response predominantly driven by COL20.

### Impact of treatment, diet, and dose of diet on serum BHB and acetate

Treatment impacted circulating BHB (*P* < 0.0001) and acetate (*P* = 0.05) concentrations (Table 2). Circulating BHB was highest in COL20 (lsmeans ± SEM; 3.23 ± 0.18 ng/μL), then COL10 (2.59 ± 0.18 ng/μL), which were not different from one another (*P* > 0.05). Circulating BHB levels were intermediate in SOS pigs, and not different from COL10, ZH (1.91 ± 0.18 ng/μL), MR20 (1.67 ± 0.18 ng/μL), and MR10 (1.75 ± 0.18 ng/μL; *P* > 0.05), but different from COL20 (Tukey *P* = 0.005). Circulating acetate was highest in ZH piglets (1.95 ± 0.15 ng/μL), and only differed from COL10 (1.24 ± 0.15 ng/μL; Tukey *P* = 0.04), which had the lowest levels of acetate. Analysis of the effect of diet, dose of diet, and the interaction on circulating BHB revealed only diet (COL vs MR) impacted circulating BHB levels (diet *P* < 0.0001) with COL20 and COL10 treatments having higher levels of BHB compared to MR20 and MR10. There was also a tendency for a diet by dose of diet interaction on circulating BHB levels (*P* = 0.07), which appears to be due to the COL20 treatment elevating BHB levels. Acetate levels were not impacted by diet, dose, or the interaction (*P* > 0.05).

### Impact of treatment, diet, and dose of diet on circulating free amino acid concentrations

Every circulating amino acid measured was altered by treatment (*P* < 0.05; Supplemental Table S11 [19]). Highlighted in the main text are impacts of treatment, diet, and dose on circulating levels of Met, Lys, Glu, Gln, Leu, and Ser. These amino acids were selected because Met and Lys were supplemented in the MR by the manufacturer, whereas Ser, Gln, and Leu concentrations were greater in pooled COL diet than MR on an as-fed basis, and Glu was one of the amino acids with the lowest concentration in pooled COL.

### Methionine

**Fig 3:**
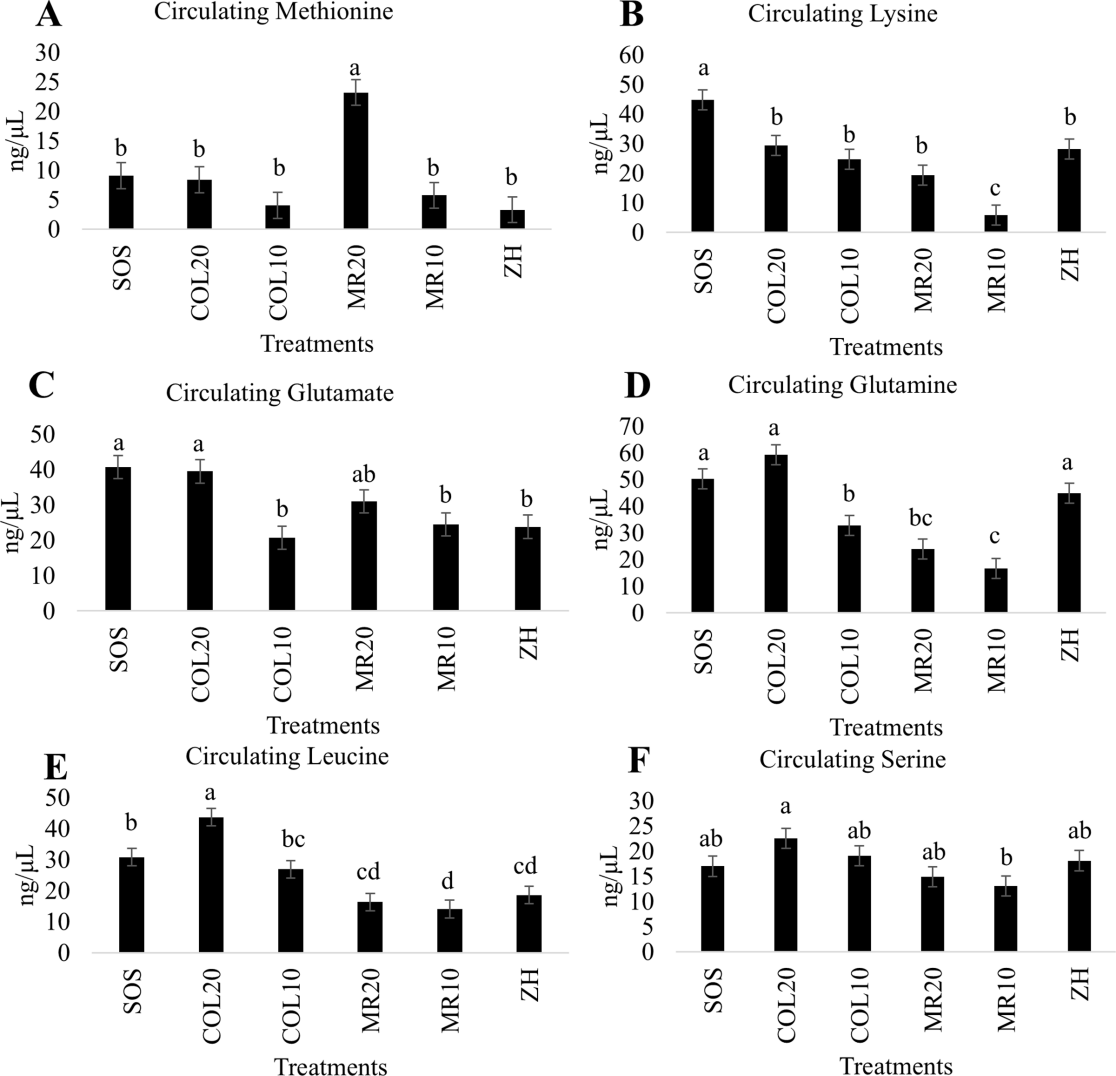
Impact of colostrum (COL) fed at 20% (COL20) or 10% (COL10) of birth body weight, milk replacer (MR) fed at 20% (MR20) or 10% (MR10) of birth body weight, *ad libitum* intake of COL by stay-on-sow (SOS), or no feeding and immediately euthanized (ZH) on circulating levels of A) methionine, B) lysine, C) glutamate, D) glutamine, E) leucine, and F) serine. Separation of means was generated by Tukey’s honest significant difference. Bars without a common letter differ (*P* ≤ 0.05).

Circulating Met was greater in piglets that consumed MR20 (lsmeans ± SEM; 23.2 ± 2.20 ng/μL) compared to all other treatments (ranged from 3.29 to 9.08 ± 2.20 ng/μL), which did not differ from each other (Fig 3A; *P* < 0.0001). Circulating Met was impacted by diet (*P* = 0.0006) and dose (*P* < 0.0001) with MR (MR20 and MR10) treatments greater than COL treatments (COL20 and COL10) and 20% (COL20 and MR20) greater than 10% (COL10 and MR10), and these responses were driven by the MR20 treatment.

### Lysine

Circulating Lys was greatest in SOS (44.7 ± 3.38 ng/μL) and lowest in MR10 (5.77 ± 3.38 ng/μL, with all other treatments intermediate (ranged from 19.4 to 29.3 ± 3.38 ng/μL), but different to these (Fig 3B; *P* < 0.0001). Circulating Lys was impacted by diet (*P* < 0.0001) and dose (*P* = 0.0001) with COL (COL20 and COL10) and 20% (COL20 and MR20) treatments greater than MR (MR20 and MR10) and 10% (COL10 and MR10), respectively.

### Glutamate

Circulating Glu was the highest in SOS (40.7 ± 3.30 ng/μL) and COL20 (39.5 ± 3.30 ng/μL), MR20 (31 ± 3.30 ng/μL) was intermediate, and COL20 and SOS differed from COL10 (20.7 ± 3.30 ng/μL), MR10 (24.5 ± 3.30 ng/μL), and ZH (23.8 ± 3.30 ng/μL; Fig 3C; *P* < 0.0001). Circulating Glu levels were almost double in 20% (COL20 and MR20) treatments compared to 10% (COL10 and MR10; *P* = 0.002), but there was no difference due to diet (*P* > 0.05).

### Glutamine

Circulating Gln was greatest in SOS (50.3 ± 3.75 ng/μL), COL20 (59.3 ± 3.75 ng/μL), and ZH (44.8 ± 3.75 ng/μL) pigs, that differed from all the other treatments with MR10 (24.5 ± 3.75 ng/μL) having the lowest circulating Gln concentration (Fig 3D; *P* < 0.0001). Circulating Gln was impacted by both diet (*P* < 0.0001) and dose (*P* < 0.0001) with levels in COL, especially COL20, more than double MR (MR20 and MR10) treatments.

### Leucine

Circulating Leu was greatest in COL20 (43.7 ± 2.85 ng/μL), differed from SOS (30.8 ± 2.85 ng/μL) and COL10 (26.9 ± 2.85 ng/μL), and MR (MR20 16.3 ± 2.85 ng/μL, MR10 14.1 ± 2.85 ng/μL) and ZH (18.6 ± 2.85 ng/μL) treatments were lowest (Fig 3E; *P* < 0.0001). Circulating Leu concentrations in MR (MR20 and MR10) treatments were approximately half to one third the levels of COL (COL20 and COL10) treatments (*P* < 0.0001) and the 20% (COL20 and MR20) treatments were greater than 10% (COL10 and MR10; *P* < 0.0001), and these responses were primarily driven by COL20.

### Serine

Circulating Ser was highest in COL20 (22.5 ± 2.00 ng/μL) and lowest in MR10 (13.1 ± 2.00 ng/μL), and all other treatments were intermediate (ranged from 14.9 to 22.5 ± 2.00 ng/μL; Fig 3F; *P* = 0.03). There was diet effect (*P* = 0.003) on circulating Ser with COL (COL20 and COL10) increasing Ser levels relative to MR (MR20 and MR10) treatments, but there was no effect of dose on circulating levels of Ser (*P* > 0.05).

### Impact of treatment, diet, dose, and diet by dose interaction on circulating lipid profiles

Of the 736 lipids considered for downstream analysis following the screening phase of MRM-profiling (1.3-fold > blank), 338 (45.9%) were TG, 72 (9.8%) DG, 136 (18.4%) PC, 19 (2.5%) PE, 16 (2.1%) PI, 4 (0.5%) PS, 19 (2.5%) PG, 8 (1.1%) Cer, 37 (5.1%) SM, 36 (4.8%) CE, and two carnitine esters (0.2%; Car). Circulating concentrations of all lipid classes measured were significantly affected by treatments (*P* < 0.05; Table 3), although treatment did not impact the ratio of TG + CE to PL + Cer (*P* > 0.05), a measure of the relative size of apolipoproteins-chylomicrons. There was a tendency for diet by dose interaction related to the circulating concentration of DG (*P* = 0.06), which was higher in COL10 (37.6 ± 2.00 AU) piglets. Analysis of the effect of diet found CE (*P* = 0.001) and PL + Cer (*P* = 0.04) greater in COL (COL20 CE 36.1 ± 4.50 AU, PL + Cer 45.8 ± 5.00 AU; COL10 CE 49.9 ± 4.50 AU, PL + Cer 52.3 ± 5.00 AU) compared to MR (MR20 and MR10 piglets. Concentration of TG was lower in COL (COL20 26.1 ± 3.40 AU, COL10 17.8 ± 3.40 AU) piglets (*P* = 0.0005) compared to MR (MR20 39.9 ± 3.40 AU and MR10 17.5 ± 3.40 AU) piglets with this response driven by MR20. Concentrations of CE and TG were impacted by dose of diet (CE *P* = 0.05 and TG *P* < 0.0001). Piglets on the 10% (COL10 and MR10) treatments had higher CE with the response driven by COL10, whereas pigs on the 20% (COL20 and MR20) treatments had higher TG with the response driven by MR20. All ZH pigs had the lowest concentrations of all lipid classes (ranged from 7.16 to 26.7 AU), except CE (20.7 ± 4.50 AU), where ZH had the second lowest concentration.

**Table 3:** Impact of colostrum (COL) fed at 20% (COL20) or 10% (COL10) of birth body weight, milk replacer (MR) fed at 20% (MR20) or 10% (MR10) of birth body weight, *ad libitum* intake of COL by stay-on-sow (SOS), or no feeding and immediately euthanized (zero hour; ZH) on concentration of circulating triacylglycerols (TG), cholesteryl esters (CE), diacylglycerols (DG), phospholipids (PL; sum of Cer, PC, PE, PI, PS, PG, SM) and TG + CE/PL + Cer ratio. Two models were used to evaluate the response variables to the effect of treatment, or the effect of diet (COL vs MR), effect of dose of food (20% vs 10%), or the interaction of diet and dose.

Lipid Class, arbitrary units	Lsmeans^1^						SEM^2^
	SOS	COL20	COL10	MR20	MR10	ZH	
DG	29.8^ab^	33.1^ab^	37.6^a^	31.9^ab^	30.8^ab^	26.7^b^	2.00
CE	21.6^b^	36.1^ab^	49.9^a^	20.0^b^	29.3^b^	20.7^b^	4.50
TG	34.2^ab^	26.1^bc^	17.8^c^	39.9^a^	17.5^c^	7.16^c^	3.40
PL + Cer	32.6^ab^	45.8^a^	52.3^a^	39.9^ab^	34.8^ab^	22.9^b^	5.00
TG + CE/PL + Cer	1.86	1.38	1.39	1.48	1.64	1.23	0.18
**Lipid Class**	**P-value**						
	**Trt**	**Rep**	**Diet**	**Dose**	Diet*dose	**Rep**	
DG	0.02	0.005	0.13	0.17	0.06	0.003	
CE	0.0001	0.07	0.001	0.05	0.99	0.05	
TG	<0.0001	0.04	0.0005	<0.0001	0.0003	0.02	
PL + Cer	0.004	0.70	0.04	0.89	0.29	0.71	
TG + CE/PL + Cer	0.19	0.01	0.65	0.92	0.88	0.02	

^1^Least square means of independent variables in response to piglets being fed different levels of COL or MR.

^2^Standard error of mean.

^(a-c)^Separation of means was generated by Tukey’s honest significant difference. Means without a common letter differ (*P* ≤ 0.05).

### Characterization of lipid profile using hierarchical cluster analysis and PCA

**Fig 4:**
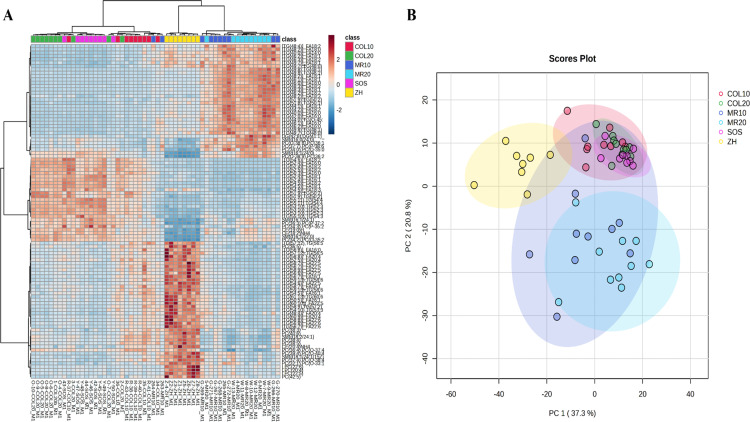
A) Heatmap with dendrogram and hierarchical clustering and B) principal component analysis 2D scores plot of circulating lipids from piglets fed colostrum (COL) at 20% (COL20) or 10% (COL10) of birth body weight, milk replacer (MR) fed at 20% (MR20) or 10% (MR10) of birth body weight, *ad libitum* intake of COL by stay-on-sow (SOS), or no feeding and immediately euthanized (ZH).

Hierarchical cluster analysis of the top 100 differential lipids revealed distinct lipid profiles for COL20 and SOS compared to MR20, MR10, and ZH (Fig 4A). COL10 exhibited unique lipid clusters that differed from MR20 and MR10 but were more closely related to ZH, COL20, and SOS. Lipids most abundant in MR20 and MR10 were absent in all other treatment groups. PCA of lipids demonstrated distinct lipid profiles in response to diet, dose of diet, and feed intake (Fig 4B). PC1 of the PCA scores plot captured greatest variance in data of 37.3%, which primarily separates ZH piglets and several dose 10% animals, from other treatments, indicating a large effect of neonate feeding on circulating lipid profiles. PC2 accounts for 20.8% of the variation in the data and primarily separates MR from COL and ZH piglets.

### Distribution of lipid profile by chain length, degree of unsaturation, and fatty acid profile of TG

For analysis of the distribution of lipids, we asked two questions: 1) What is the effect of adequate intake of COL (SOS or COL20) or MR (MR20) compared to birth (ZH) on the distribution of circulating lipids and 2) What is the effect of diet (COL20, COL10 vs MR20, MR10) and dose of diet (COL20,MR20 vs COL10, MR10) on the distribution of circulating lipids? Analysis of the response to adequate intake of MR (MR20) and adequate COL (SOS and COL20) compared to ZH found 289 lipids differed significantly between MR20 and ZH, and 377 lipids differed significantly between adequate COL (SOS and COL20) and ZH (FDR < 0.05). Analysis of the impact of diet revealed that 449 lipids differed between COL and MR treatments, whereas 196 lipids were significantly affected by dose (FDR < 0.05).

### Impact of adequate intake of COL vs ZH on distribution of chain length and degree of unsaturation

**Fig 5:**
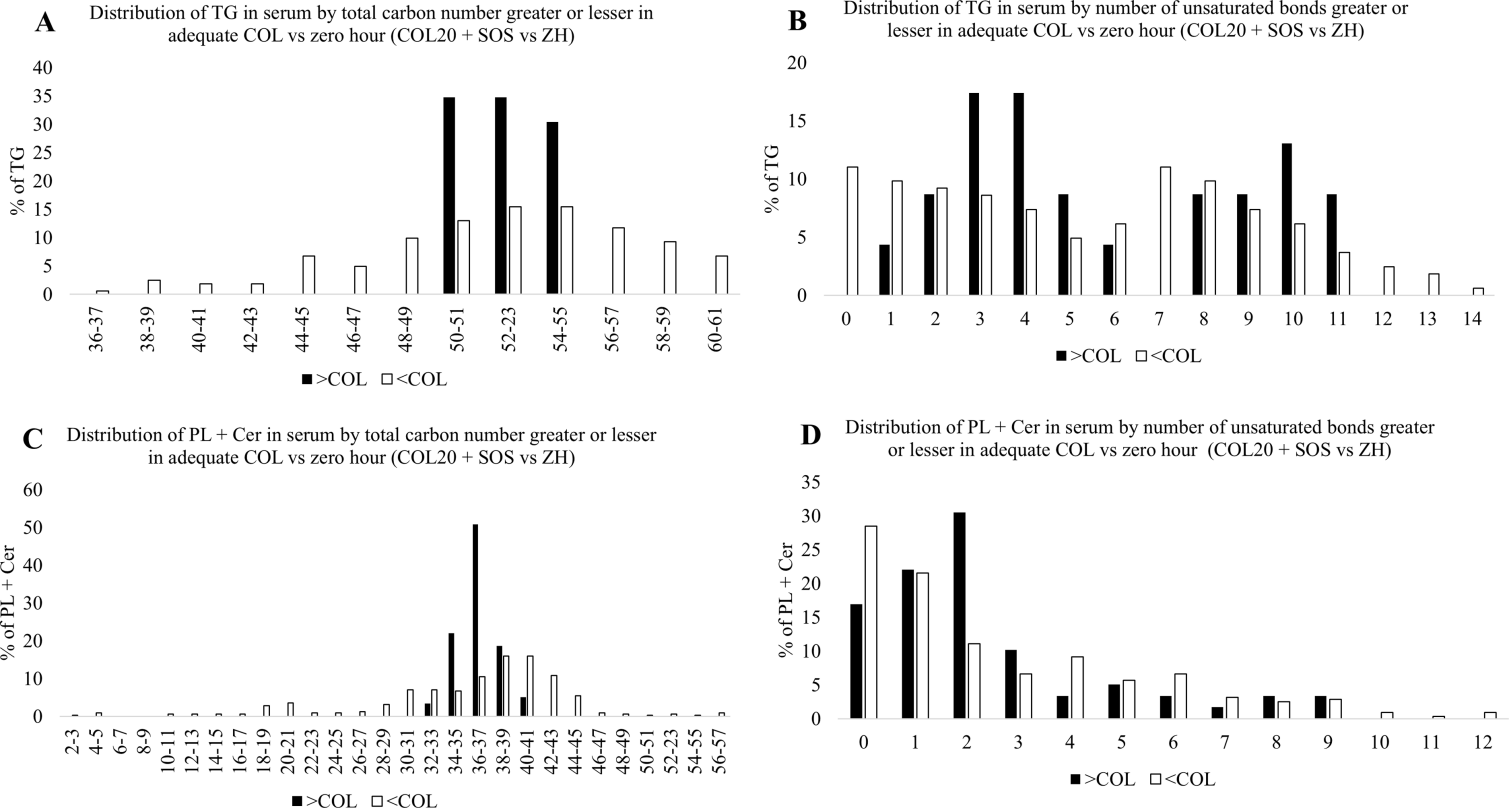
Impact of adequate colostrum (COL) intake (SOS + COL20) relative to zero hour (ZH) piglets on profile of circulating triacylglycerols (TG) and phospholipid + ceramides (PL + Cer) total carbon length (TG: Fig 5A; PL + Cer: Fig 5C) and number of unsaturated bonds (TG: Fig 5B; PL + Cer: 5D). Black bars represent percentage greater in COL-fed animals and white bars represent percentage lesser in COL-fed animals.

Analysis of the TG class of lipids differentially abundant between piglets with adequate COL (SOS and COL20) and ZH piglets (Fig 5), found adequate COL (SOS and COL20) increased the distribution of TG with carbon chain lengths between 50 and 55, while ZH piglets had a greater distribution of TG with longer carbon chains, ranging between 57 and 61, as well as shorter chains, ranging between 36–49 (Fig 5A). Additionally, adequate COL (SOS and COL20) increased the percentage of TG with 3–5, 10, and 11 unsaturated bonds, whereas ZH had a lower proportion of TG with saturated fatty acids and a greater proportion of TG with 1, 7, and 12–14 unsaturated bonds (Fig 5B). Adequate COL (SOS and COL20) increased the proportion of PL + Cer with carbon chain lengths of 34–37, while PL + Cer profile of ZH had longer chain lengths of 40–57 and shorter chains from 33 and below (Fig 5C). Adequate COL (SOS and COL20) also increased the percentage of PL + Cer with 2 unsaturated bonds, while ZH had a greater proportion of PL + Cer with 0, 4 and 6 unsaturated bonds as well as those with 10–12 unsaturated bonds (Fig 5D). Thus, adequate COL compared to circulating profiles at birth altered the distribution of PL + Cer by reducing both carbon chain length and the degree of unsaturation in PL + Cer.

### Impact of adequate intake of MR vs ZH on distribution of chain length and degree of unsaturation

**Fig 6:**
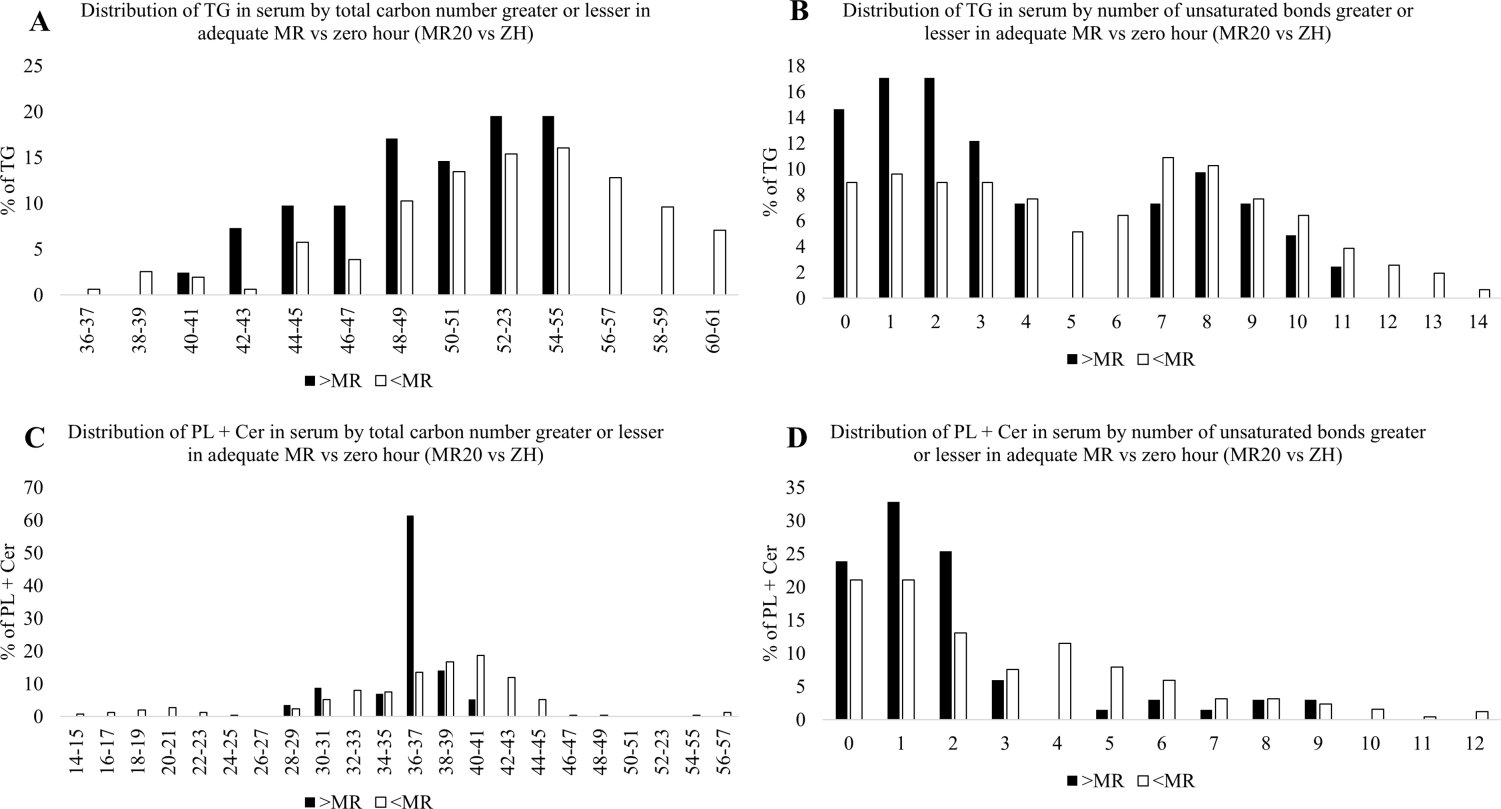
Impact of adequate milk replacer (MR) intake (MR20) relative to zero hour (ZH) piglets on profile of circulating triacylglycerols (TG) and phospholipid + ceramides (PL + Cer) total carbon length (TG: Fig 6A; PL + Cer: Fig 6C) and number of unsaturated bonds (TG: Fig 6B; PL + Cer: 6D). Black bars represent percentage greater in MR-fed animals and white bars represent percentage lesser in MR-fed animals.

Adequate MR (MR20) compared to ZH increased the percentage of TG with shorter carbon chain lengths, ranging from 42 to 55, while ZH piglets had TG with longer chain lengths, from 56 to 61 (Fig 6A). Additionally, MR20 increased the percentage of TG with lower unsaturation levels (1–3 unsaturated bonds), whereas ZH had a greater abundance of TG with moderate to high unsaturation (5–7 and 12–14 unsaturated bonds; Fig 6B). Piglets fed MR20 had increased proportion of PL + Cer with total carbon chain lengths of 36–37 carbons, while ZH had PL + Cer with longer carbon chains, ranging from 40 and above, as well as shorter chains from 24 and below (Fig 6C). Adequate MR (MR20) increased PL + Cer with 1–2 unsaturated bonds, whereas ZH had PL + Cer with greater unsaturated bonds between 4–6 and 11–12 (Fig 6D).

### Impact of diet on distribution of chain length and degree of unsaturation

**Fig 7:**
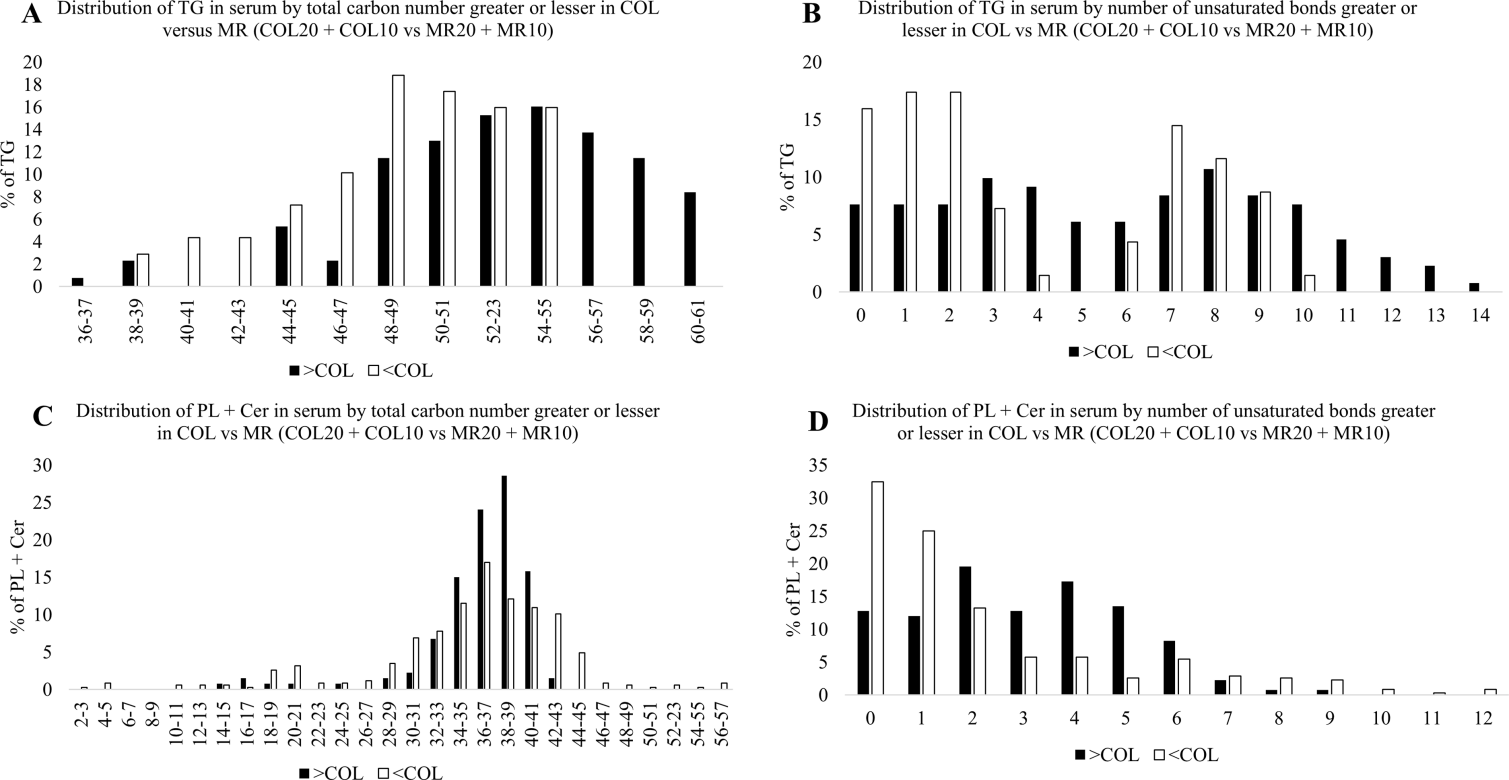
Impact of colostrum (COL; COL20 + COL10) versus milk replacer (MR; MR20 + MR10) feeding on profile of circulating triacylglycerols (TG) and phospholipid + ceramides (PL + Cer) total carbon length (TG: Fig 7A; PL + Cer: Fig 7C) and number of unsaturated bonds (TG: Fig 7B; PL + Cer: 7D). Black bars represent percentage greater in COL-fed animals and white bars represent percentage lesser in COL-fed animals.

Analysis of the effect of diet (COL20, MR20 vs COL10, MR10) on circulating lipid profiles found when compared to MR (MR20 and MR10), COL (COL20 and COL10) had a higher percentage of TG with longer carbon chain lengths (56–61), while MR (MR20 and MR10) had a greater percentage of TG with shorter carbon chains containing 40–51 carbons (Fig 7A). Piglets that consumed COL (COL20 and COL10) treatments also had a greater percentage of TG with unsaturation levels containing 3–6 and 10–14 unsaturated bonds, whereas MR (MR20 and MR10) had a greater proportion of TG with less unsaturation containing 0–2 and 7 unsaturated bonds (Fig 7B). Within PL + Cer, COL (COL20 and COL10) increased the percentage of carbon chains with 34–41 carbons while MR (MR20 and MR10) promoted longer carbon chains greater than 42. Piglets that consumed COL (COL20 and COL10) treatments also increased the percentage of PL + Cer with higher unsaturation containing 2–6 unsaturated bonds, while the MR (MR20 and MR10) piglets had PL + Cer with lower unsaturation containing 0–1 unsaturated bonds (Fig 7C and 7D).

### Impact of dose of diet on distribution of chain length and degree of unsaturation

**Fig 8:**
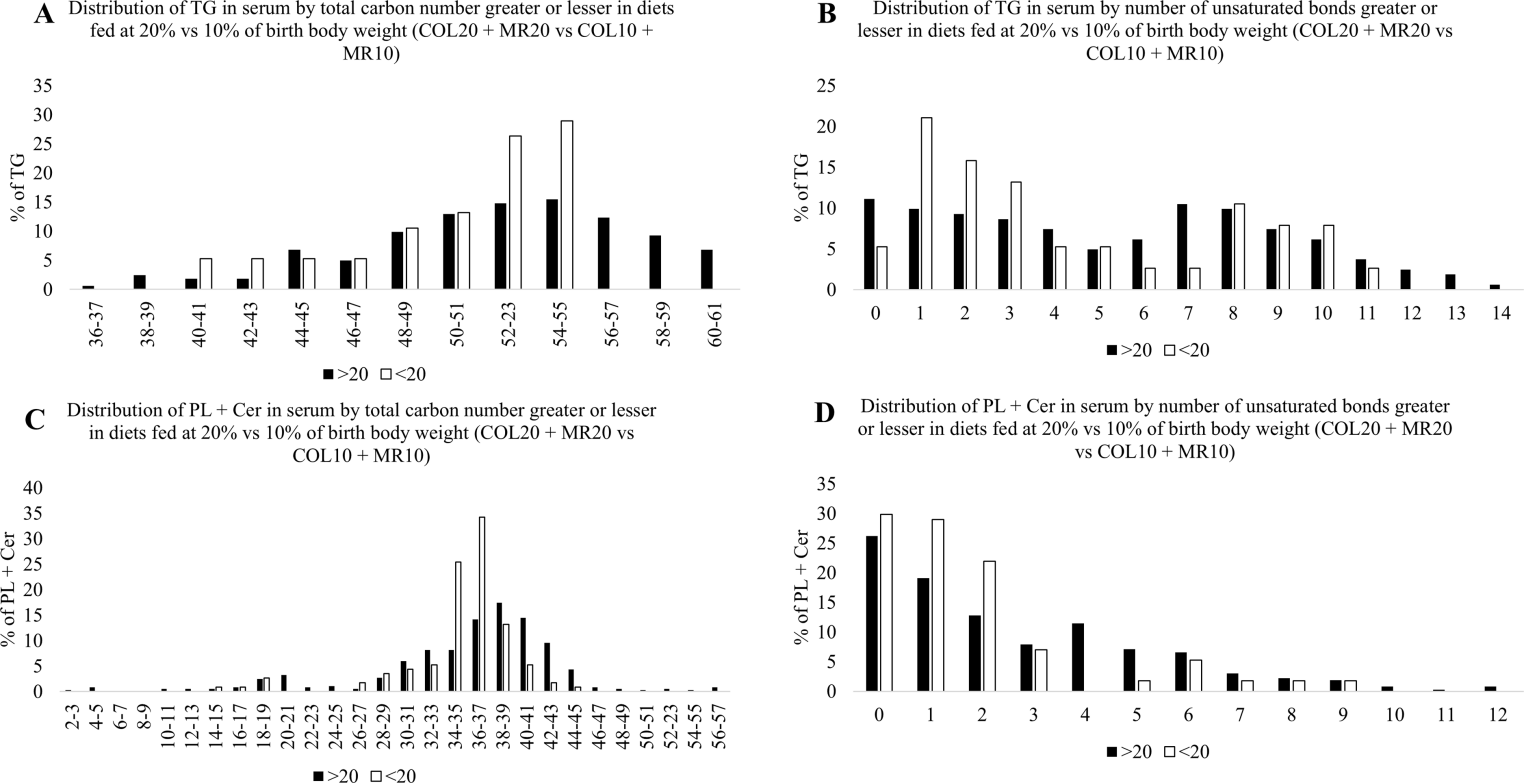
Impact of higher dose of food (COL20 + MR20) versus lower dose of food (COL10 + MR10) on profile of circulating triacylglycerols (TG) and phospholipid + ceramides (PL + Cer) total carbon length (TG: Fig 8A; PL + Cer: Fig 8C) and number of unsaturated bonds (TG: Fig 8B; PL + Cer: 8D). Black bars represent percentage greater in higher dose of food (20%) and white bars represent percentage lesser in higher dose of food (20%).

A higher nutrient dose (20%; COL20 and MR20) increased the percentage of TG with carbon chains greater than 56 carbons while the lower dose (10%; COL10 and MR10) favored TG with shorter chains containing ≤ 50 carbons (Fig 8A). Piglets on 20% (COL20 and MR20) treatments had increased percentage of TG with 5–6 and 10–14 unsaturated bonds, while piglets on 10% (COL10 and MR10) treatments had increased percentage of TG with lower unsaturation levels containing 0–2 and 7 unsaturated bonds (Fig 8B). For PL + Cer, piglets on the 20% (COL20 and MR20) treatments demonstrated higher percentage of PL + Cer with carbon chains between 34–41 carbons, whereas piglets on the 10% (COL10 and MR10) treatments promoted PL + Cer with chain lengths of 18–21, 26–31, and above 42 carbon chain length (Fig 8C). Piglets on the 20% (COL20 and MR20) treatments demonstrated increased percentage of PL + Cer with 2–6 unsaturated bonds, whereas piglets on the 10% (COL10 and MR10) treatments demonstrated increased percentage of PL + Cer with unsaturation 0–1 and 8–12 unsaturated bonds (Fig 8D).

### Impact of adequate COL and MR vs ZH, diet, and dose on fatty acid profile of TG

**Fig 9:**
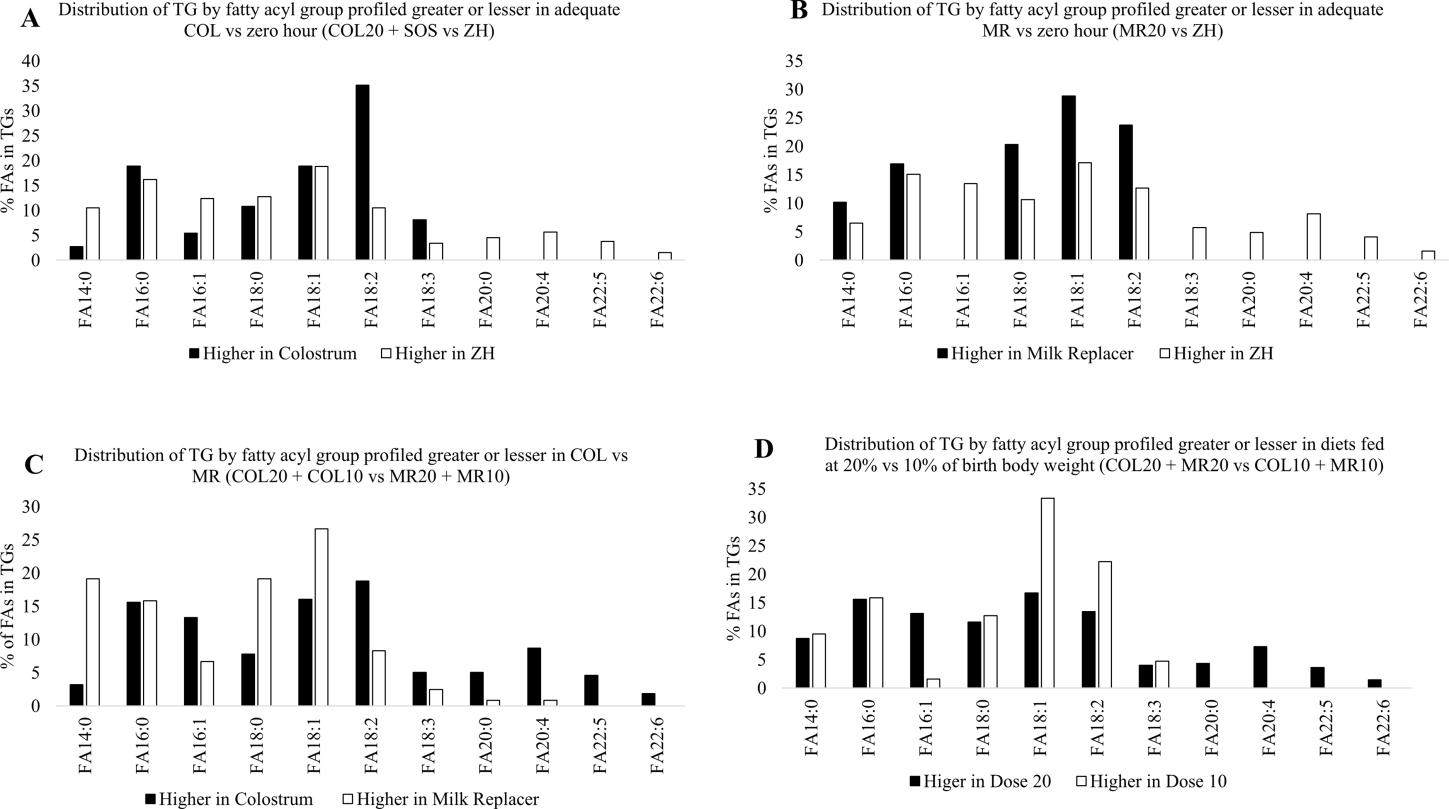
Impact of adequate colostrum (COL; COL20 + SOS; Fig 9A) or milk replacer (MR; MR20; Fig 9B) versus zero hour (ZH), COL (COL20 + COL10) versus MR feeding (MR20 + MR10; Fig 9C), and higher (COL20 + MR20) versus lower level (COL10 + MR10) of food (Fig 9D) on the distribution of fatty acyl group from triacylglycerols (TG) in circulation of neonatal piglets. Black bars represent the percentage of fatty acids from TG greater in adequate COL vs ZH, adequate MR vs ZH, COL vs MR, or 20% vs 10%. White bars represent the percentage of fatty acids lesser in COL vs ZH, adequate MR vs ZH, COL vs MR, or 20% vs 10%.

Analysis of the fatty acid profile of TG revealed TG containing FA18:2 were more abundant in adequate COL (SOS and COL20) compared to ZH, while TG with FA20:0, FA20:4, FA22:5, and FA22:6 were more prevalent in ZH than in adequate COL (SOS and COL20; Fig 9A). Adequate MR (MR20) piglets had higher levels of TG with FA14:0, FA18:0, FA18:1, and FA18:2 compared to ZH. However, TG containing FA18:3, FA20:0, FA20:4, FA22:5, and FA22:6 were more abundant in ZH than in MR20 piglets (Fig 9B). Analysis of the effect of diet showed that piglets that received COL (COL20 and COL10) had a greater proportion of TG with FA 18:2, FA18:3, FA20:0, FA20:4, FA22:5, and FA22:6 than MR (MR20 and MR10), whereas TG containing FA14:0, FA18:0 and FA18:1 were more prominent in MR (MR20 and MR10) than in COL (COL20 and COL10; Fig 9C). Analysis of the effect of dose (COL20, MR20 vs COL10, MR10) showed piglets on the 20% (COL20 and MR20) treatments had higher levels of TG with FA16:0 and FA20:0, FA 20:4, FA22:5, and FA22:6, whereas piglets on the 10% (COL10 and MR10) treatments had TG with more FA18:1 and FA18:2 (Fig 9D).

### Impact of adequate COL or MR compared to ZH, and diet and dose of diet on circulating metabolite profile

**Fig 10:**
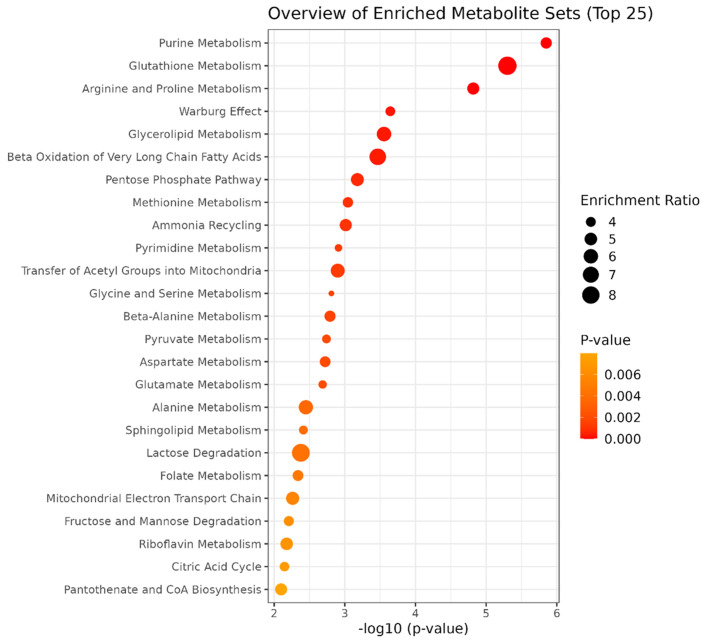
The top 25 metabolic pathways significantly enriched with 57 metabolites more abundant in milk replacer (MR) than colostrum (COL). The enrichment ratio is determined by the size of the circle and the p-value is determined by the range of red (*P* < 0.0001) to orange/yellow (*P* = 0.006). Pathways with darker red and larger circles are more significantly enriched than pathways with smaller, orange/yellow circles.

Out of 324 negatively ionized metabolites identified during the screening phase, 67 metabolites exhibited signal intensities at least 1.3-fold greater than the blank after normalization by sum of ion intensities and were selected for further analysis. Similarly, of 371 positively ionized metabolites, 156 were 1.3-fold above the blank and were included in downstream analysis. The linear model applied to the negative ion dataset using dose (COL20 and MR20 vs COL10 and MR10) as the primary factor and diet (COL20 and COL10 vs MR20 and MR10) as a covariate, revealed a significant effect of dose on only one metabolite, pantothenic acid (FDR < 0.05). Analysis of the positive ion dataset found diet impacted 92 MRM corresponding to 63 unique metabolites (FDR < 0.05; Supplemental Table S19 [19]). Piglets fed COL (COL20 and COL10) had six metabolites more abundant than MR (MR20 and MR10), whereas MR had 57 metabolites greater than COL fed animals. Among these, 6 metabolites were more abundant in COL-fed piglets: L-Proline, L-Tyrosine, L-Phenylalanine, Valine, Succinic acid, 4-Hydroxy-L-proline with 5 of the 6 metabolites being amino acids. Pathways enriched with the 57 metabolites elevated in MR (MR20 and MR10) compared to COL (COL20 and COL10) treatments included purine metabolism, glutathione metabolism, Warburg effect (NADP; NADPH; Oxaloacetic acid; ATP; D-Ribulose 5-phosphate; Glyceraldehyde 3-phosphate; GDP; FAD; ADP), and lactose degradation (Fig 10). Metabolites elevated in MR (MR20 and MR10) treatments relative to COL (COL20 and COL10) also notably included methionine, xanthine, hypoxanthine, and bilirubin (Supplementary Table S19). The comparisons between adequate COL (COL20 and SOS) and ZH and adequate MR (MR20) and ZH did not reveal metabolites that were significantly different.

## Discussion

Varying levels of COL intake over the first 24 h postnatal relate to differences in long-term growth, production performance, and fertility of swine [1,26]. The overall goal of this study was to better understand the role of colostrum-specific components (diet) versus the amount of nutritional intake (dose) on circulating immunocrit, protein, glucose, BHB, and acetate levels as well as amino acid, lipid, and small metabolite profiles. In general, we found that both diet (COL20 and COL10 vs MR20 and MR10) and dose of diet (COL20 and MR20 vs COL10 and MR10) affected circulating levels of nutrients and metabolites. Data also demonstrated that although COL source (pooled COL versus SOS samples) varies in composition, adequate intake of this first milk similarly impacted circulating lipids, nutrient and metabolite profiles, and responses were distinct from adequate intake of MR (MR20), supporting a role for COL in shaping early life metabolic responses.

### Despite colostrum and milk replacer diets differing in gross energy, lactose, protein, and fat content, piglets on similar doses demonstrated similar 24 h weight gain

The gross energy content and relative level of macronutrients was greater in COL than MR, with gross energy content of the pooled COL approximately 1 kcal/g higher. Despite differences, COL20 and MR20 gilts gained a similar amount of weight over the 24 h feeding period. We believe the level of lactose in MR promoted the growth of the MR20 piglets as the lactose content was four times greater in MR (12%) than pooled COL (3%). Lactose is a disaccharide of glucose and galactose. Exploratory metabolite data indicated higher circulating levels of D-galactose in MR treatment groups, likely reflective of higher lactose intake. Circulating levels of glyceraldehyde-3-phosphate (G3P), glycerol, oxaloacetate, and glucogenic amino acids, glycine and serine, were also higher in MR fed piglets compared to COL. These metabolites along with ATP, ADP, GDP, FAD, NADP/NADPH and D-Ribulose 5-phosphate enriched the Warburg effect pathway, which potentially reflects a state of selective aerobic glycolysis. Aerobic glycolysis results in the accumulation of glycolytic intermediates, which leads to the production of nucleotides, amino acids, and fatty acids, ultimately, promoting cellular proliferation [29], which may be promoting the growth evident in the MR20 neonates. G3P is a glycolytic intermediate and sits at the intersection of glycerol breakdown to support gluconeogenesis or utilization by the TCA cycle. Piglets on MR treatments demonstrated greater circulating levels of glycerol, which may reflect substrate available for glucose synthesis or energy generation via the TCA cycle. Oxaloacetate also sits at a critical junction between the TCA cycle and gluconeogenesis by supporting the first committed step of gluconeogenesis and continued cycling of the TCA cycle. Increased levels of these glycolytic intermediates in serum may be an indicator of glycolytic status of MR fed animals, and the animal’s dependence on glycolytic pathways for energy generation.

We believe the lack of difference between COL20 and MR20 circulating glucose levels is due to mechanistically different reasons. Firstly, circulating glucose concentration in the MR20 treatment could be due to three reasons: 1) lactose content of the MR diet breaking into glucose and galactose; 2) increased supply of gluconeogenic substrates like glycerol and OAA, as demonstrated by the exploratory metabolite analysis; or 3) ability of piglets to utilize galactose to supply glycolytic intermediates through entry of glycolysis at glucose-6-phosphate sparing the use of glucose, and ultimately, contributing to higher circulating levels of glucose. Secondly, circulating glucose concentration in the COL20 treatment could be due to glucose sparing, because an alternative energy source, such as fats, is being prioritized for use. This idea is supported by lower circulating TG levels in COL20 piglets, suggesting either their breakdown into fatty acids and glycerol for use, or fat storage as TG. Increased circulating BHB concentrations in COL20 piglets support TG catabolism over storage because BHB is a by-product of fat breakdown, as its synthesis is derived from acetyl CoA, the end product of β-oxidation. Findings indicate that the response of MR piglets is in contrast with the metabolic transition that occurs in response to COL intake, which stimulates pathways needed for lipid metabolism to include peroxisome biogenesis and fatty acid oxidation [8,17,30].

The higher gross energy content measured in the pooled COL versus MR samples was primarily due to the much higher protein content of COL. The protein content in MR consists mainly of whey and casein proteins. Milk protein in swine MR is derived from bovine mature milk as there are no commercial swine-based milk replacers or colostrum replacers. Immunoglobulin proteins were the most abundant type of protein in MR, which was expected, as relative to COL, the proportion of immunoglobulins as milk proteins is lower in mature milk. In addition, during the processing of milk to produce MR, immunoglobulins may be degraded and potentially rendered inactive [31]. Despite presence of immunoglobulins in MR diet, immunocrit levels were not different between ZH and MR treatments. Others that reported feeding bovine derived MR to neonatal piglets for the first two days postnatal found minimal changes in circulating levels of IgG [32] potentially because immunoglobulins are denatured and rendered inactive during the processing of milk into MR. Immunocrit level of COL10 animals was similar to MR20, MR10, and ZH immunocrit responses, demonstrating a dose response to standardized level of pooled COL and consistent with our previous reports [18]. Despite immunocrit of COL10 animals being lower than SOS and COL20, circulating protein levels were similar among SOS, COL20, and COL10, potentially reflecting that colostral proteins other than immunoglobulins are being absorbed across the small intestine in neonates. At birth, fetal-type enterocytes line the piglet’s gut, and appear to be capable of taking up entire proteins and other macromolecules via vacuolization and transmitting the whole proteins into circulation with factors in COL potentiating this process [12,13]. Protease inhibitor proteins are high in COL (Table S3) [14,22] and when combined with the more basic pH of the immature gut, prevents hydrolyzation of proteins [33] together facilitating the maintenance of colostral protein structure and potentially their activity in the neonate’s system. Given the nuances of protein metabolism of the neonatal piglet gut, we believe COL fed animals are not reliant on protein to support growth at this stage. We posit lipids serve as the primary energy source in the COL diet, rather than proteins or lactose, which is supported by findings of others that demonstrate COL intake initiates peroxisomal biogenesis and fatty acid oxidation in neonates [8,29].

### Exploratory metabolite analysis indicates maintenance of colostrum fat bioactivity post-absorption

In support of the supposition that protein from COL maintains bioactivity following absorption is the finding that circulating metabolites elevated in MR20 and MR10 compared to COL20 and COL10 piglets included hypoxanthine, xanthine, bilirubin, and acylcarnitine. Elevated hypoxanthine and xanthine levels in MR20 and MR10 piglets suggest lower xanthine dehydrogenase/oxidase (XDH/XO) activity [34]. XDH/XO is a major milk fat globule membrane protein, that metabolize hypoxanthine/xanthine, [35], and defects lead to elevated circulating levels of xanthine [36]. Thus, a higher circulating levels of xanthine in the MR treatment support a potential role of COL-derived XDH/XO protein and its activity. Also, higher bilirubin concentrations in MR treatments potentially reflect altered heme metabolism and impaired liver function in these animals. α-casein, κ-casein and lactoferrin function as heme-binding proteins [37], and lower concentration and inactivity of these proteins in MR compared to those in pooled COL could contribute to the higher concentration of circulating bilirubin in the MR group [38]. Likewise, higher circulating levels of acylcarnitine in MR may suggest incomplete mitochondrial or peroxisomal fatty acid oxidation as acylcarnitine is a product of β-oxidation and increased levels are a marker of mitochondrial dysfunction [39]. Greater circulating levels of acylcarnitine could also explain the increased concentration of TG in MR animals, particularly MR20.

### Circulating levels of amino acids were influenced by dietary content of amino acids, as well as colostrum-specific components

Circulating concentrations of Met, Leu, Gln, and Ser likely reflected the amount of these free amino acids in the pooled COL and MR diets. However, although dietary Lys and Glu were highest in MR diet, COL (COL20 and COL10) piglets had higher circulating concentrations of Lys and Glu. Amino acids utilize amino acid transporters to be absorbed across the gut. Glu transport is mediated by the apical transporter excitatory amino acid transporter 3 (EAAT3) encoded by solute transporter *SLC1A1* [40,41], whereas Lys is transported by *SLC6A19*, cationic amino acid transporter (*CAT1*), or Linker for Activation Of T Cells Family Member 1 (*LAT1*) [42]. Studies demonstrated that COL feeding initiates the activity of amino acid transporters in the small intestine of neonatal piglets [43]. The finding that dietary levels of Glu and Lys did not reflect circulating levels in respective treatments, may be due to potentiating effects of COL on their transport/absorption. Glu is a primary fuel for enterocytes, and so lower levels in MR (MR20 and MR10) piglets may reflect Glu catabolism in the small intestine [44]. Studies also reported that supplemental Lys fed to week-old piglets increased villus height, mucosa depth, and overall enhanced gut integrity [45], suggesting that it may be primarily metabolized by enterocytes, and post absorption levels are affected by variable factors. Therefore, when interpreting these data, it is important to consider that circulating levels of amino acids reflect the combination of absorption and post absorption metabolism. Glu is glucogenic and can be used to support glucose production or to supply TCA cycle intermediates in other organs. Lys could be shunted toward ketone production. Therefore, the differential response of dietary Glu and Lys to circulating Glu and Lys could be linked to small intestine amino acid transporter activity, and distinct metabolic pathways turned on in the small intestine and liver of COL (COL20 and COL10) and MR (MR20 and MR10) piglets. Future studies are needed to understand the relationship between differential circulating amino acid profiles and the metabolic response of neonates to amino acid diet content as well as the influence of the level of intake.

### Circulating lipid profiles were influenced by feeding and adequate colostrum intake and lipid concentrations reflect adequate nutrient levels

Lipid distribution in piglets was markedly influenced by feed intake. The distinct clustering pattern of the ZH group compared to piglets fed COL or MR highlights the metabolic impact of early postnatal feeding in shaping circulating lipid profiles. The progressive disappearance of specific lipid species with increasing COL and MR intake likely reflects enhanced metabolic utilization and clearance through pathways such as mitochondrial β-oxidation, lipoprotein lipase–mediated hydrolysis, and tissue uptake [46]. Colostrum specific components may promote a metabolic shift of certain lipid profiles identified in ZH piglets toward efficient lipid oxidation and incorporation into tissues. Lipid profiles were similarly influenced by diet with distinct lipid clusters between COL (COL20 and COL10) and MR (MR20 and MR10) groups, suggest that COL specific components modulate lipid metabolism differently from MR diet. These differences may reflect divergent impacts on lipid digestion, absorption, and subsequent metabolic processing as well as the make-up of lipids in diets.

Elevated levels of long-chain, polyunsaturated TG in circulation of ZH piglets, likely reflecting the in-utero transfer of essential fatty acids from the sow during gestation, consistent with reports of passive placental transfer of long-chain polyunsaturated fatty acids in swine [47]. Greater feed intake maintained the presence of long-chain and more unsaturated TG in circulation, suggesting enhanced substrate availability for lipid metabolism and the potential for long-term metabolic programming.

Lard and coconut oil served as the primary source of fat in MR (Supplemental Table S1 [19]). The ratio of saturated to unsaturated fats is 40:60 in lard, and 90:10 in coconut oil [48]. Approximately 50% of the fatty acids in coconut oil are medium chain lengths (6–12 carbons) and approximately 92% of fats in lard are long chain fatty acids (13–21 carbons in length) [49,50]. The TG profile (measured using MRM profiling) of pooled COL and MR diets closely reflected the circulating TG profile of piglets in the COL (COL20 and COL10) and MR (MR20 and MR10) treatments, respectively. In particular, the circulating abundance of saturated, medium-chain TG in MR (MR20 and MR10) piglets could be derived from coconut oil in MR diet.

## Conclusion

In the first 24 h postnatal, the primary drivers of weight gain, circulating glucose, and regulation of rectal temperature appear to be adequate level of nutrient intake regardless of source of nutrients. Circulating profiles of amino acids, small metabolites, and lipids as well as protein concentration and immunocrit were affected by both the dose of food and diet composition. Despite COL having a much higher solid (dry matter) and energy content than MR, the COL and MR animals that received adequate intake (SOS, COL20, MR20) gained a similar amount of weight. Colostrum had a much higher protein content than MR, whereas lactose levels were much higher in the MR diet. Differences in circulating profiles of protein between the COL and MR treatments indicated that colostral proteins may remain intact with bioactivity maintained in post-absorption. Moreover, the high lactose content of MR appeared to promote greater dependence on glycolytic pathways to support growth of MR-fed animals, whereas COL-fed animals may more readily oxidize fat compared to MR-fed animals. Future studies are needed to mechanistically evaluate how COL specific components alter metabolism and nutrient utilization of organs, and how differences in dietary nutrients mediate circulating profiles to influence development and growth in the neonatal piglet.

## Supporting information

S1 File Checklist. (PDF)
